# Fluorination of Bidimensional Materials

**DOI:** 10.3390/ma19194252

**Published:** 2026-10-08

**Authors:** Abderraouf Rouag, Tara Buzdon, Guillaume Haddad, Mostafa S. Eraky, Yasser Ahmad, Batiste Clavier, Nicolas Batisse, Kevin Lemoine, Laurent Jouffret, Katia Guérin, Fabrice Leroux, Pierre Bonnet, Marc Dubois

**Affiliations:** 1 Institut de Chimie de Clermont-Ferrand (UMR 6296), Université Clermont Auvergne, CNRS, 24 Avenue Blaise Pascal, 63178 Aubière, France; abderraouf.rouag@uca.fr (A.R.); tara.buzdon@uca.fr (T.B.); batiste.clavier@uca.fr (B.C.); nicolas.batisse@uca.fr (N.B.); kevin.lemoine@uca.fr (K.L.); laurent.jouffret@uca.fr (L.J.); katia.araujo_da_silva@uca.fr (K.G.); fabrice.leroux@uca.fr (F.L.); pierre.m.bonnet@uca.fr (P.B.); 2 Batteries Technology Department, Central Metallurgical Research and Development Institute (CMRDI), Helwan P.O. Box 87, Cairo 11421, Egypt; mostafa.shawky@uca.fr; 3 Department of Renewable Energy Engineering, College of Engineering, Fahad Bin Sultan University, Tabuk 47721, Saudi Arabia; yahman@fbsu.edu.sa

**Keywords:** two-dimensional materials, fluorine chemistry, topochemical fluorination, plasma fluorination, polymer-mediated fluorination, defect engineering

## Abstract

Fluorination has emerged as a powerful strategy for tailoring the structure, chemistry, and electronic properties of two-dimensional (2D) materials beyond conventional surface functionalization. This review highlights fluorination chemistry as a versatile toolbox for the controlled design of layered materials, with particular emphasis on synthesis approaches, reaction mechanisms, and structural transformations of graphene and carbon materials, hexagonal boron nitride (h-BN), transition-metal dichalcogenides (TMDs), MXenes, layered double hydroxides (LDHs), and layered metal oxides. Various fluorination methods, including direct fluorination using F_2_, XeF_2_ treatment, HF-based processes, plasma-assisted fluorination, electrochemical routes, hydrothermal/solvothermal methods, and polymer-mediated topochemical fluorination, are critically discussed regarding their mechanisms, advantages, limitations, and ability to control fluorine content, bonding configuration, defect formation, and phase evolution. Finally, current challenges related to fluorine control, scalability, environmental impact, and advanced characterization are discussed. Fluorination–defluorination chemistry is thus presented as a promising platform for atomic-scale engineering of 2D materials.

## 1. Introduction

Beyond graphene and related carbon materials, fluorination appears as a versatile strategy for tailoring the structure and properties of a broad range of two-dimensional (2D) materials. These include h-BN, TMDs such as MoS_2_ and WS_2_, MXenes, LDHs, and layered metal oxides. Owing to their possibly large specific surface area, highly accessible surface atoms, and stacking more or less expanded and anisotropic structures, 2D materials provide numerous platforms for controlled fluorination reactions capable of modifying both surface chemistry and/or bulk properties [1,2]. A wide variety of fluorination routes have been developed, including gas/solid and liquid/solid processes. Elemental fluorine (F_2_) remains one of the most powerful fluorinating agents, enabling direct fluorination over a broad range of temperatures and fluorine contents [3]. Alternative approaches include XeF_2_, HF-derived reagents, NF_3_, CF_4_, SF_6_-based plasmas, fluoropolymers acting as fluorine sources, hydrothermal and solvothermal fluorination, electrochemical fluorination, and plasma-assisted treatments [4,5]. On the other hand, plasma fluorination enables surface-selective functionalization, defect engineering, and patterning. Gas/solid and plasma fluorination processes are particularly attractive because they avoid solvent contamination and allow homogeneous treatment of powders, nanosheets, films, and multilayer assemblies [6]. The incorporation of fluorine deeply changes the structural, electronic, optical, magnetic, and chemical properties of 2D materials. The fluorination can also be limited to the surface in order to change only the surface chemistry and energy or add/suppress active sites. Depending on the host lattice and fluorination rate, fluorine may induce bandgap opening, modulation of carrier concentration, changes in magnetic behavior, enhanced hydrophobicity, improved oxidation resistance, modified interlayer interactions, and increased thermal or chemical stability [7]. Moreover, in layered compounds, fluorination can facilitate exfoliation by weakening interlayer forces, stabilize metastable phases like diamane, or modify ion transport pathways [8]. In MXenes and layered oxides, fluorine frequently acts as a surface termination species controlling conductivity, electrochemical performance, and interfacial reactivity [9]. In TMDs, fluorination can tune semiconducting properties, catalytic activity, and phase stability, while in h-BN it provides a route to modify the electronic structure of an intrinsically wide-bandgap insulating material [10].

The technological applications of fluorinated 2D materials are quickly growing. In the energy domain, fluorinated carbon materials are broadly employed as cathodes in lithium/fluorocarbon batteries, where strong C–F bonds support high energy density [11,12]. Furthermore, fluorination provides a productive strategy to adjust the electronic and optical properties of 2D materials [13,14]. For example, fluorinated graphene enables bandgap engineering of graphene, while fluorinated MoS_2_ and WS_2_ display improved carrier transport and optical responses, supporting applications in nanoelectronics, optoelectronics, and sensing devices. Owing to their low surface energy and chemical stability, fluorinated layered materials such as fluorinated graphene and fluorinated h-BN have revealed promising tribological performance as solid lubricants and low-friction coatings. Additional applications comprise protective anti-corrosion coatings, separation membranes based on fluorinated graphene oxide and MXenes, and dielectric materials with tunable electrical properties. These varied applications highlight fluorination as a powerful chemical modification strategy for expanding the functionality of 2D materials.

Several reviews have addressed the fluorination or halogenation of 2D materials. Previous reviews have included halogen functionalization of 2D materials, including fluorine, chlorine, bromine, and iodine [15], while others have predominantly explored on fluorination of graphene and associated carbon materials, highlighting C–F bonding, fluorination extent, synthesis routes, and resulting properties [16]. More recent studies have assessed specific fluorination approaches, such as XeF_2_-assisted fluorination and etching of graphene, h-BN, and TMDs [4], or gas-phase fluorination of h-BN for multifunctional applications [17].

In contrast, the present review focuses specifically on fluorination chemistry across chemically diverse 2D material families, including carbonaceous materials, h-BN, TMDs, MXenes, LDHs, and layered oxides. It systematically connects the fluorinating agent and processing route to fluorine incorporation, bonding configuration, structural evolution, fluorination–defluorination mechanisms, and resulting physicochemical properties. Particular attention is given to how the chemical nature and structure of the host regulate the balance between surface functionalization, bulk fluorination, interlayer modification, exfoliation, phase transformation, and competing decomposition. This comparative framework emphasizes both common fluorination principles and material-specific behavior, providing an integrated view of fluorination mechanisms and structure–property relationships across diverse classes of 2D materials.

Fluorination is not the only way to modify the properties of 2D materials. Elemental doping and substitution can effectively modulate their electronic, magnetic, and optical characteristics, while defect engineering offers additional control over band structure and functionality. Recent studies have revealed, for example, that Si substitution can tune the electronic and magnetic properties of graphene nanoribbons [18], whereas edge configuration, defects, and stacking can regulate the electronic properties of silicene nanoribbons [19,20]. These complementary strategies highlight the broad tunability of 2D materials and provide an important context for understanding the distinctive role of fluorination in modifying their surface chemistry, bonding, and electronic structure.

Accordingly, this review provides a comparative, fluorination-centered analysis of representative 2D material families, including carbonaceous materials, h-BN, TMDs, MXenes, LDHs, and layered oxides. Selected recent and representative studies are examined to show how the nature of the 2D host, fluorinating agent, reaction conditions, and fluorination degree govern fluorine incorporation, bonding configuration, structural evolution, and resulting properties. Particular attention is given to materials with highly accessible surfaces, such as graphene and related carbon nanosheets, and highly reactive systems such as h-BN, MXenes, and layered oxides, where fluorination may involve a balance between functionalization, structural modification, and decomposition. The review discusses the mechanisms of fluorine incorporation, migration, bonding, and removal, supported by experimental studies and Density Functional Theory (DFT) calculations that provide insights into activation barriers, transition states, and bond energies [21,22,23,24,25,26,27,28]. This comparative approach identifies common fluorination principles and material-specific behaviors across chemically distinct 2D systems and highlights their technological relevance in energy storage, catalysis, sensing, tribology, insulators, and spintronics.

The materials discussed in this review are presented following a progression that reflects both the historical development of the field and the increasing complexity of fluorination chemistry. We begin with carbonaceous materials, which constitute the most mature class of fluorinated 2D systems and provide the fundamental concepts governing fluorination mechanisms, structure–property relationships, and fluorination–defluorination processes. h-BN is then considered as a structurally related material exhibiting a high probability of forming gaseous BF_3_ and NF_3_ during fluorination (i.e., decomposition). MXenes and TMDs illustrate how fluorination can modify surface terminations, electronic structure, and phase stability in more complex layered compounds. Finally, LDHs and layered oxides are discussed as representative ionic layered materials, highlighting the diversity of fluorine incorporation mechanisms encountered in 2D systems. This progression provides a coherent framework for understanding fluorination across a broad range of 2D materials.

## 2. Carbonaceous Materials

### 2.1. Gas/Solid Fluorination Mechanisms via F_2_

**Figure 1:**
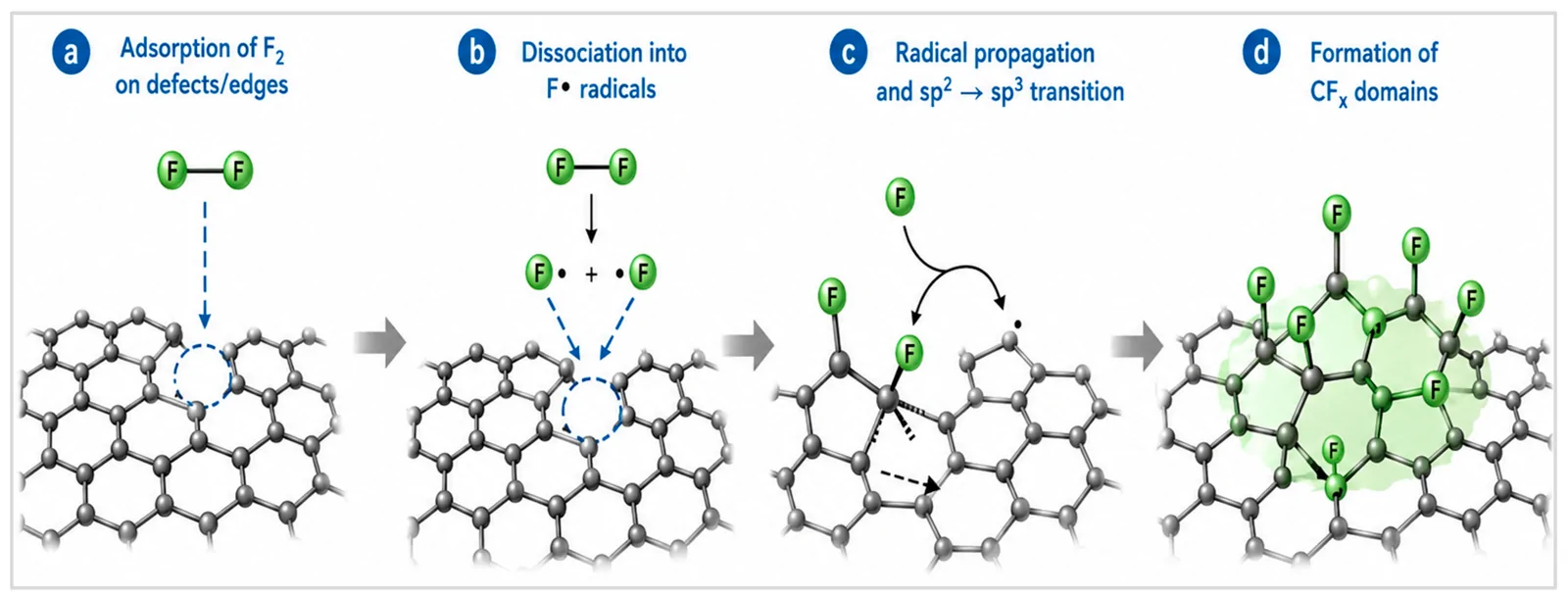
Radical fluorination mechanism of graphene: (**a**) $F_{2}$ adsorption at defect/edge sites, (**b**) dissociation into $F^{\cdot }$ radicals, (**c**) radical propagation causing local ${sp}^{2}\to {sp}^{3}$ hybridization, and (**d**) growth of fluorinated ${CF}_{x}$ domains.

Gas/solid fluorination of 2D carbon materials by elemental fluorine F_2_ is a heterogeneous process that begins with the initial activation of the fluorine molecule, followed by a radical chain reaction on the surface of the carbon network (Figure 1) [29,30]. Under standard conditions (ambient temperature to 200 °C), the adsorption of F_2_ on a graphene surface is low, and the dissociation of the molecule is kinetically unfavorable due to its high bond energy (158 kJ/mol). An increase in temperature (200–600 °C) is therefore necessary to overcome this barrier and initiate the reaction [31].

**Figure 2:**
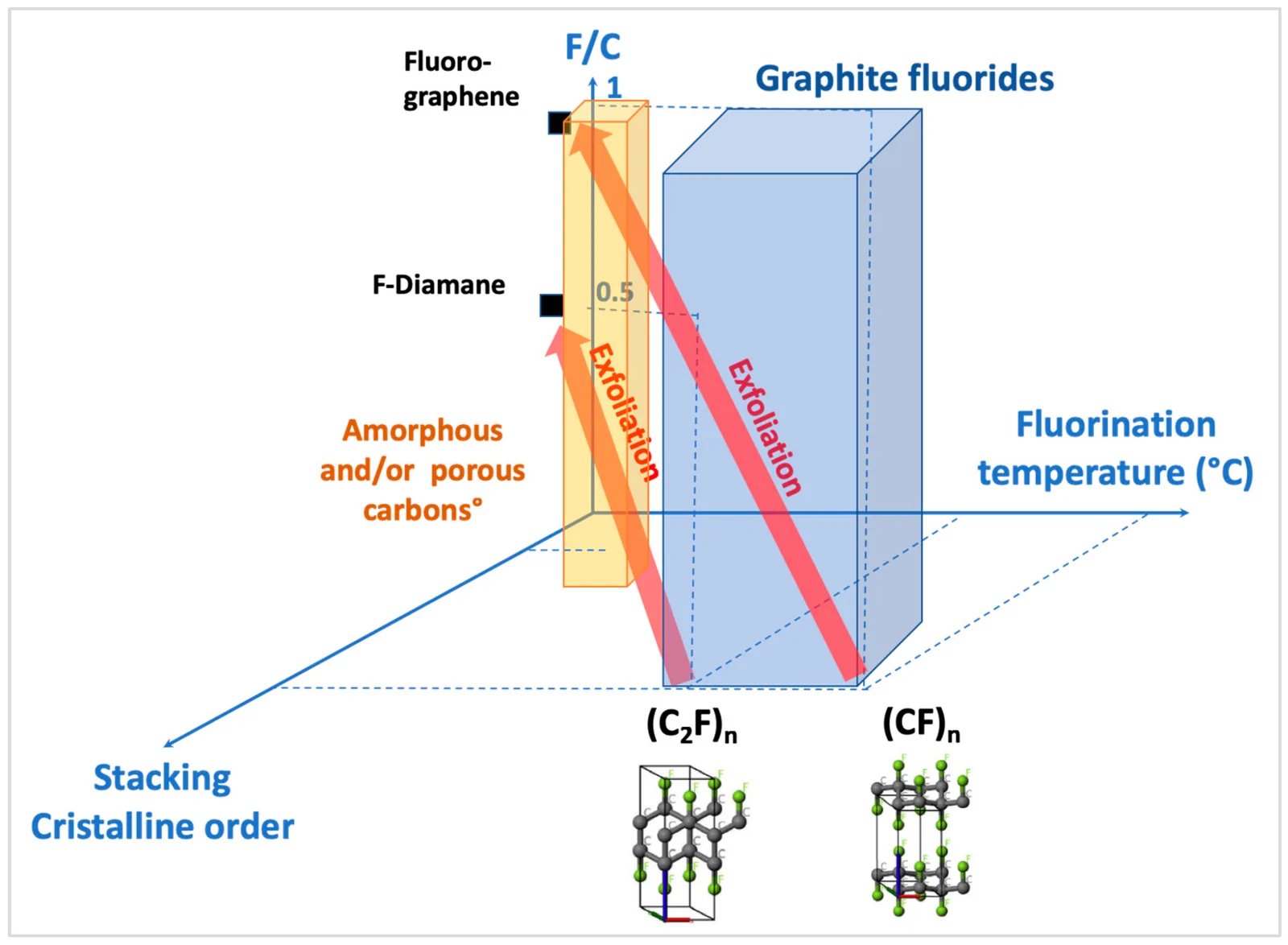
Schematic representation of main parameters controlling carbon fluorination: crystalline stacking order, synthesis temperature, and stoichiometry (F/C ratio).

The initiation step occurs preferentially at reactive sites such as structural defects like vacancies, sheet edges, or oxygen-containing functional groups (epoxides, hydroxyls), which facilitate the homolytic dissociation of F_2_ into F^•^ radicals and the formation of the first C–F bonds [29,30,32]. Precise spectroscopic measurements show that the energy is 154.7962 ± 0.0048 kJ mol^−1^ (1.6040 ± 0.00005 eV) [33]. Once initiated, fluorination propagates via a radical mechanism: the addition of a fluorine atom to an sp^2^ carbon induces a local transition to sp^3^ hybridization, which disrupts the π system and increases the reactivity of neighboring carbons [34]. This local activation promotes successive fluorine additions, leading to the formation of CF_x_-type fluorinated domains. The process gradually becomes autocatalytic, as the presence of fluorine alters the electron density and lowers the adsorption and dissociation barriers for new F_2_ molecules [35]. As fluorination progresses, competition may arise between the formation of C–F bonds and the degradation of the carbon network, particularly in highly exfoliated or highly reactive materials, where volatile fluorinated products (CF_4_, C_2_F_6_) may be generated [36]. The nature of C–F bonds changes with the degree of fluorination [29,35]. At low fluorine content (F/C < 0.3), the bonds exhibit a partially ionic or semi-ionic character, interacting with the residual sp^2^ regions. This interaction is evidenced by shifts in the XPS (C1s peak at ≈289 eV), infrared (1100 cm^−1^), and NMR (80–88 ppm for ^13^C nuclei) spectra [37,38,39,40,41,42,43]. This structural evolution leads to a significant change in electronic properties, with a gradual transition from a conductive or semiconducting material to insulating behavior (electronic bandgap up to 3–4 eV for F/C ≈ 1) [30,40,42]. The F/C ratio is a key parameter for characterizing these transformations [13] (Figure 2): (i) with low fluorination (F/C ≈ 0.05–0.3), the material largely retains its sp^2^ lattice, and the C–F bonds remain dispersed and often semi-ionic. These materials are used for applications requiring residual conductivity, such as sensors and flexible electronics [13]. (ii) At intermediate levels (F/C ≈ 0.3–0.7): A mixture of sp^2^ and sp^3^ regions is observed, with both covalent and semi-ionic bonds coexisting. This hybrid structure is utilized for battery electrodes and supercapacitors [44], (iii) with high fluorination ratio (F/C ≈ 0.8–1): The material approaches stoichiometric phases of the (CF)_n_ structural type, characterized by a predominance of C–F covalent bonds and an entirely sp^3^ structure. These materials are used as cathodes in Li/CF_x_ primary batteries and as solid lubricants [45], see thereafter.

### 2.2. Structural Parameters Influencing Fluorination

#### 2.2.1. Crystalline Order

The crystalline order of 2D carbon materials plays a decisive role in gas-solid fluorination, as it governs the initiation kinetics, the spatial distribution of C–F bonds, and the resulting F/C atomic ratio. As illustrated in Figure 2, the tridimensional schematic representation highlights the interdependent structural and processing parameters controlling the gas-solid fluorination of 2D carbonaceous materials. The orthogonal axes define the key intrinsic and extrinsic factors, namely fluorination temperature (°C), stacking/crystalline order of the graphitic host matrix, and the resulting fluorine-to-carbon (F/C) atomic ratio.

As shown, pristine, highly ordered three-dimensional graphite frameworks enable controlled fluorine intercalation and diffusion at elevated temperatures (typically 350–650 °C), leading to the formation of well-ordered bulk graphite fluorides such as stage-2 (C_2_F)_n_ or stage-1 (CF)_n_, characterized by strong covalent C–F bonding. In contrast, structural exfoliation routes that reduce interlayer stacking and enhance surface accessibility (orange vertical domain) significantly lower both thermodynamic and kinetic barriers for F_2_ dissociation. This structural transition facilitates a rapid transformation from the sp^2^ carbon framework to highly distorted sp^3^ configurations at substantially reduced temperatures.

In highly crystalline materials (well-ordered graphite, high-quality CVD graphene), the sp^2^ lattice is extensive and defect-free, resulting in low initial reactivity toward F_2_. Fluorination then begins primarily at the edges of the layers or at rare point defects, and propagation is slower and more homogeneous [46]. The bonds formed gradually evolve from semi-ionic to covalent as the fluorination rate increases. The F/C ratio remains initially low (≈0.05–0.3), but can reach high values close to 1 (phase (CF)_n_) under severe conditions (T > 500 °C, prolonged time) [41,42,47]. Conversely, in disordered materials (reduced graphene, amorphous carbons, exfoliated graphites), the high defect density promotes the adsorption and dissociation of F_2_ [48]. The reaction is faster but also more heterogeneous, leading to a non-uniform distribution of fluorinated groups. The F/C ratio is generally intermediate (≈0.2–0.7), as the presence of defects limits the formation of perfectly stoichiometric structures and sometimes promotes side reactions (etching, CF_4_ formation) [40,48,49]. The fluorination with molecular fluorine of carbonaceous, and more generally 2D materials, is inherently associated with highly exothermic reactions due to the combination of the relatively low dissociation energy of the F–F bond (154.8 kJ mol^−1^) and the formation of strong covalent or ionic C–F, B–F, or M–F bonds. Under poorly controlled conditions, particularly when the temperature is increased too rapidly or when high F_2_ partial pressures and gas flow rates are employed, the rate of heat generation may exceed the capacity of the system to dissipate the released energy. In such cases, local thermal runaway phenomena may occur, resulting in over-fluorination, structural degradation, loss of crystallinity, excessive defect generation, or even partial decomposition of the host material. For carbonaceous materials, this may lead to C–C bond cleavage, carbon loss through formation of volatile fluorocarbons, and collapse of the graphene framework. Careful control of fluorination conditions is therefore essential. Progressive temperature ramps, dilution of F_2_ in inert gases, moderate fluorine flow rates, and stepwise fluorination protocols are commonly employed to balance fluorine incorporation and heat release.

#### 2.2.2. Stacking

**Figure 3:**
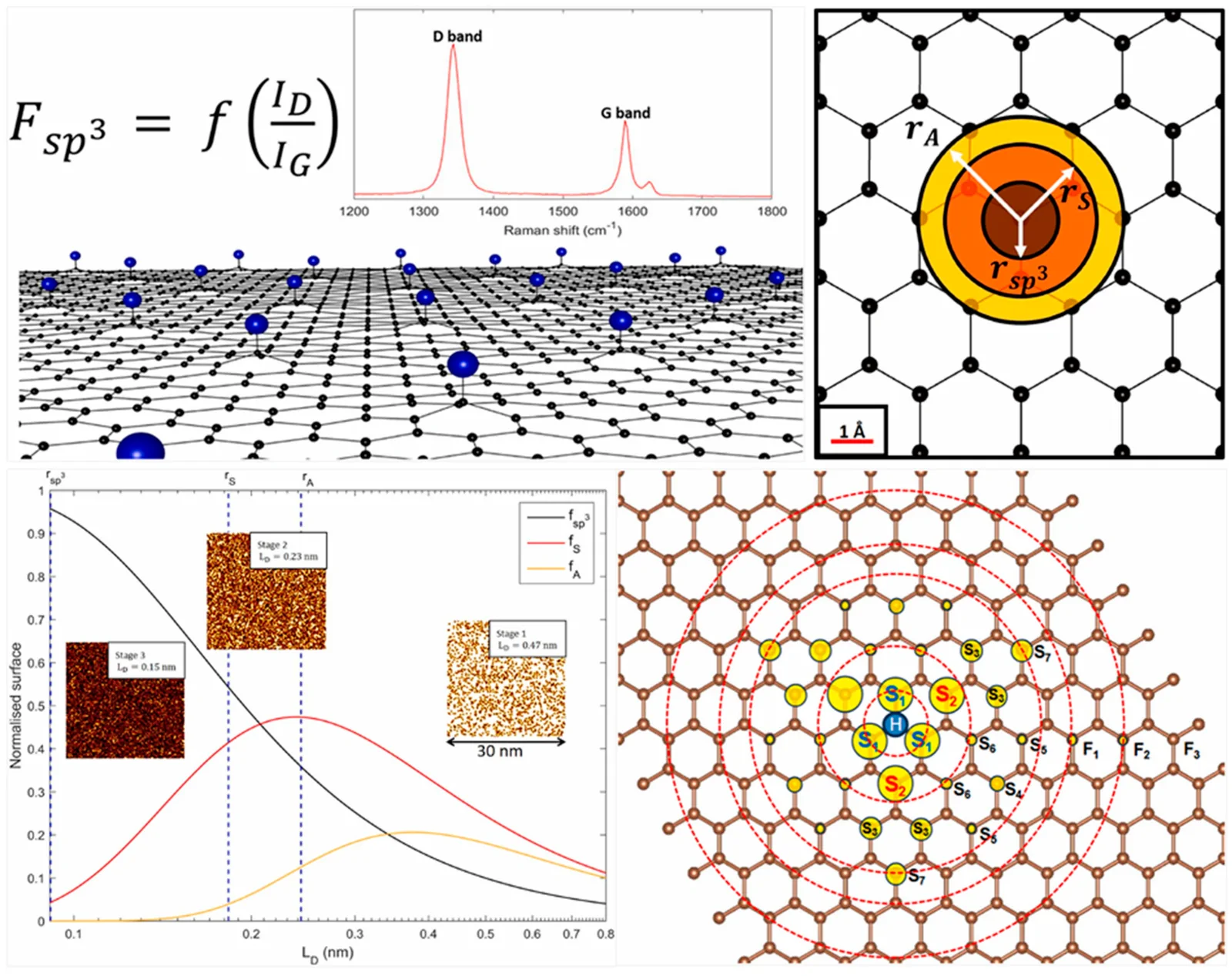
Methodology for determining ${sp}^{3}/{sp}^{2}$ ratios in functionalized graphene using Raman spectroscopy (IDIG), depicting defect radius models $\left(r_{{sp}^{3}}{,\;r}_{S}{,r}_{A}\right)$ and surface fraction evolution versus defect distance $\left(L_{D}\right)$ [52]. Adapted with permission from [52].

The stacking of layers directly controls gas/solid fluorination by F_2_ by modulating surface accessibility, fluorine diffusion, and the stability of fluorinated structures [6]. Fluorination with F_2_ of 2D carbon materials depends heavily on the number of layers and the stacking mode, as these parameters control the accessibility of reactive surfaces, the diffusion of fluorine between layers, and the symmetry of functionalization [50]. They therefore directly influence the reaction kinetics as well as the distribution of C–F bonds within the structure. In the case of an isolated graphene monolayer, both faces of the lattice are fully accessible to fluorinating species, allowing for symmetric, rapid, and homogeneous fluorination. The absence of interlayer constraints promotes efficient conversion of the sp^2^ lattice to sp^3^, leading to the formation of stoichiometric fluorographene (CF)_n_ under appropriate conditions (low and even room temperature). F_2_ gas is often diluted for milder conditions. Etching away of graphene may occur [51]. These results reveal the importance of atmospheric water in the synthesis chemistry. Under these conditions, the F/C ratio can reach 1, corresponding to near-complete saturation of carbon sites by strong covalent C–F bonds, without internal diffusion limitations. Fluorination is a powerful strategy for tuning the structure and properties of 2D carbon materials by gradually converting sp^2^ carbon networks into more sp^3^-like frameworks. Intermediate fluorination enhances performance by balancing structural distortion and active site creation to prevent restacking because it maintains conductivity while avoiding insulating states, which optimizes material efficiency for high-performance applications in chemical sensing, electrocatalysis, and energy storage (Equation (1)) (Figure 3) [52].

(1) $$f\left(\sigma \right)=1-\exp\left(-{\pi r}^{2}\sigma \right)$$

This evolution can be described in terms of the fraction of sp^3^ carbon and is commonly observed using Raman spectroscopy through changes in the D/G intensity ratio and peak broadening (Equation (2))

(2) IDIG α f(σ,rSrAS)

For bi- or few-layer systems with weak or turbostratic stacking, surface accessibility remains relatively high but becomes asymmetric between outer and inner layers [53]. The binding energy of F on bilayer systems is calculated to be slightly smaller than on monolayers [53]. The surface layers fluorinate first, while the inner layers require gradual diffusion of fluorine across the interlayer interfaces. This constraint introduces slower kinetics and structural heterogeneity, characterized by a fluorination gradient across the thickness of the material. This results in the coexistence of covalent C–F bonds at the surface and more polarized or semi-ionic bonds in the partially fluorinated regions. The fluorination ratio remains intermediate, typically ranging from 0.3 to 0.8, depending on the number of layers and reaction conditions [35,36]. In the case of AB-stacked multilayer graphite, fluorine access to the inner layers is strongly limited by van der Waals interactions between sheets, with a cohesive energy of approximately 2 eV, which stabilizes the stack and reduces the system’s permeability. Fluorination therefore begins primarily at the outer surfaces and edges, before slowly progressing inward via combined mechanisms of fluorine intercalation and diffusion. This process is significantly slower and requires more severe conditions—typically several hours at 350–650 °C—to achieve a high degree of functionalization. Under these latter conditions, the F/C ratio can approach 1, leading to the formation of relatively ordered (CF)_n_-type compounds, corresponding to nearly complete fluorination of the accessible graphitic network.

#### 2.2.3. Lateral Size

The lateral size of the sheets influences fluorination by determining the density of reactive edges and the diffusion distance of fluorine [35,54]. Overall reactivity is therefore not solely a function of chemical composition, but also of the system’s actual dimensionality. In the case of nanosheets (ranging from a few nm to <1 µm), the proportion of atoms located at the edges is extremely high. These sites exhibit significantly increased chemical reactivity due to the presence of dangling bonds, local stresses, and intrinsic structural defects. This high density of active sites significantly lowers the activation energy barrier for F_2_ dissociation, enabling rapid fluorination even at relatively low temperatures (≈200 °C). Once initiated, the reaction propagates efficiently across the entire sheet, particularly when both faces are accessible, frequently leading to highly fluorinated materials with a high F/C ratio (≈0.6–1) [55]. For sheets of intermediate size (≈1–10 µm), the proportion of edge sites decreases significantly, which alters the overall fluorination kinetics. In this regime, reaction initiation is still dominated by the edges and structural defects, but fluorine propagation becomes the limiting factor. The radical mechanism then leads to the gradual growth of fluorinated domains that extend from the edges toward the center of the sheets. This spatial diffusion of the fluorination front results in partially functionalized materials, with an intermediate F/C ratio, typically ranging from 0.3 to 0.7. Finally, in the case of large sheets (>10 µm), the edge density becomes very low, less than 1%, which makes the initiation of fluorination much more difficult. Under these conditions, the reaction is mainly limited to accessible surfaces and rare internal defects. The dissociation of F_2_ then requires more energetic conditions, with temperatures reaching up to 300–600 °C, to enable the activation of less reactive sites. The initial fluorination ratio is therefore low (≈0.05–0.3), but can gradually increase to values close to 1 after prolonged or more aggressive treatments, depending on the exposure time and the diffusion of fluorine within the lattice.

#### 2.2.4. Hetero-Elements (O, N, S, B, etc.)

The presence of heteroatoms in 2D carbon materials plays a role in fluorination by F_2_, as it modifies both the local electron density, the distribution of reactive sites, and the stability of the bonds formed. Hetero-elements act on fluorination by introducing electronic and structural perturbations [42,43,44,45,46].

These effects lead to distinct mechanisms depending on the nature of the heteroatom (O, N, or B), with direct consequences on the degree of fluorination and the resulting surface chemistry. In the case of oxygen, particularly in graphene oxide (GO), the presence of epoxide, hydroxyl, and carbonyl groups introduces both structural defects and highly reactive electrophilic centers. These groups promote the adsorption and dissociation of F_2_ by locally lowering the reaction energy barriers [56]. Fluorination may then be accompanied by substitution or epoxide ring-opening processes, leading to the coexistence of C–F bonds and residual oxygen-containing functional groups. The resulting material is therefore chemically highly heterogeneous, with an F/C ratio that can vary between 0.3 and 0.9, depending on the initial degree of oxidation and the availability of reactive sites. A two-step fluorination of GO results in an island structure of aromatic regions in a continuous phase of fluorine regions. A new electronic band gap structure matches the maximum excitation wavelength; then numerous excitons are generated, which is a precondition for strong fluorescence emission [57].

**Figure 4:**
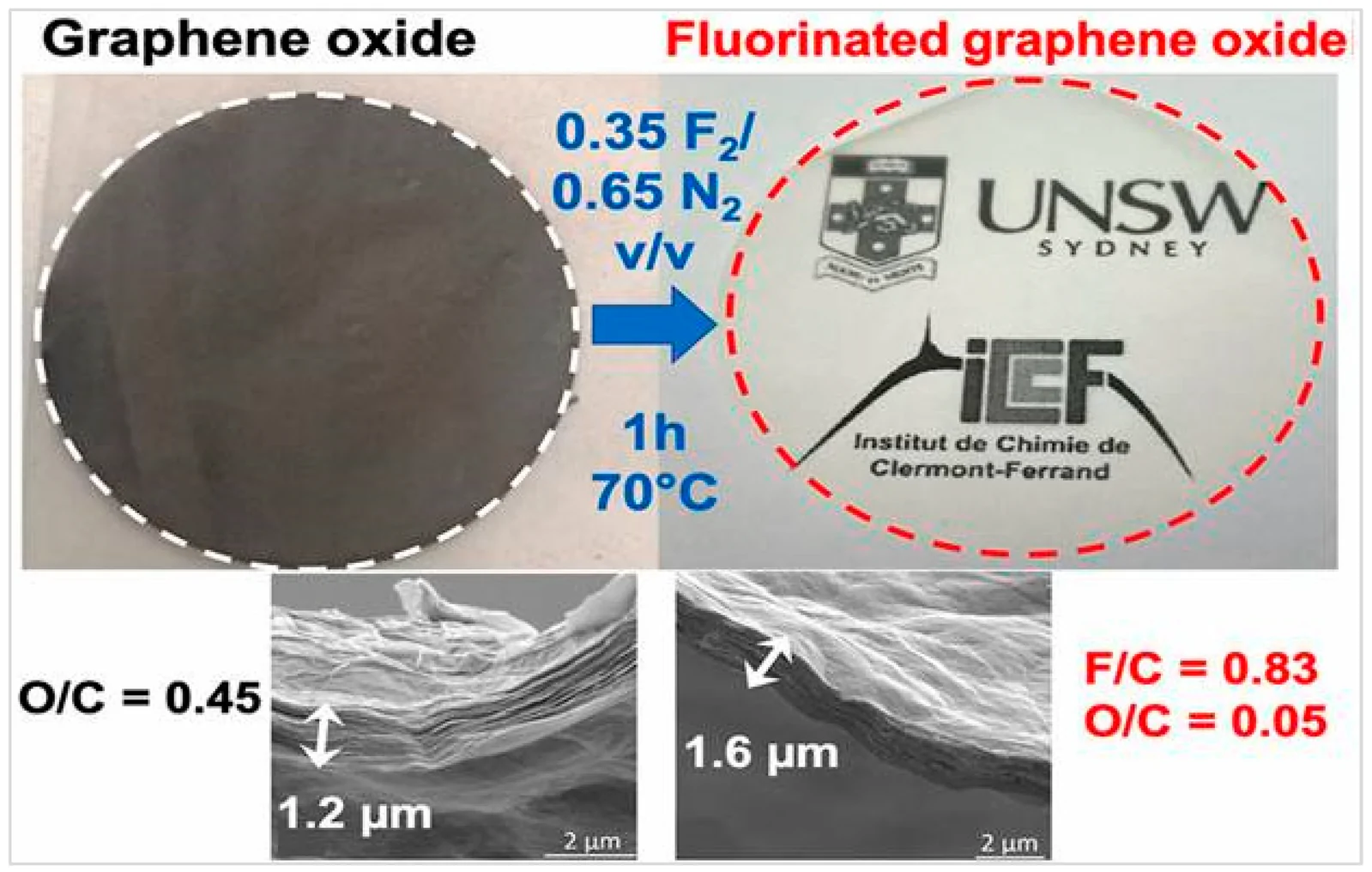
Direct synthesis of transparent fluorinated graphene oxide (FGO) films via gas-phase fluorination of graphene oxide (GO), illustrating changes in visual transparency, atomic composition (FC and OC ratios), and film thickness; figure adapted from [59].

GO was also fluorinated at room temperature using fluorine-nitrogen mixed gas in different ratios [7,58]. Fluorination of GO membrane increases hydrophobicity, lowers surface energy, and improves chemical stability. These combined structural and physicochemical changes allow fluorinated graphene films to sustain transparency and mechanical robustness while presenting tunable electronic properties for applications in flexible electronics, protective coatings, and membrane technologies (Figure 4) [59].

Defects produced by an oxygen plasma have little effect on the reactivity of monolayer graphene. On the contrary, in bilayer graphene, reactivity decreases at low defect densities but increases when defect density is higher [60]. KOH pre-activation of needle coke generates numerous structural defects and oxygen functional groups, which significantly enhance carbon–fluorine reactivity and substantially reduce the fluorination temperature [61].

The incorporation of fluorine and oxygen creates polarized carbon domains and sp^2^/sp^3^ hybridized regions, which strengthen Li^+^ affinity and support uniform nucleation. Fluorine-oxygen co-doped graphene was then utilized as an effective interfacial layer for stabilizing lithium metal anodes via the regulation of lithium deposition behavior. This porous, defect-rich architecture facilitates rapid ion transport and reduces current density hotspots, effectively restraining dendrite growth. Furthermore, the fluorinated surface promotes a stable, LiF-rich SEI, reducing electrolyte consumption and interfacial resistance. Consequently, the system accomplishes high Coulombic efficiency and low voltage hysteresis, enabling a safer, high-performance pathway for advanced lithium-metal battery technologies through accurate heteroatom engineering [62].

For nitrogen-doped materials, the situation is different because pyridinium, pyrrole, or graphitic nitrogen modifies the electronic density of the sp^2^ lattice and introduces basic sites capable of stabilizing reaction intermediates during F_2_ activation. This electronic modulation promotes a more controlled and generally more homogeneous fluorination, dominated by the formation of well-defined covalent C–F bonds. The fluorination ratio remains intermediate, typically ranging from 0.2 to 0.8, depending on the concentration and configuration of the nitrogen atoms in the lattice [63]. Mild fluorination conditions prevent severe decomposition in F_2_ of porous N-doped carbons and limit loss of specific surface area. Micropores, which host most catalytic sites, are most affected by fluorination.

In the case of boron doping, the effect is the opposite: boron introduces an electron deficiency into the carbon lattice, creating sites that strongly attract fluorinating species. This increased polarization promotes a more direct interaction with F_2_ and can lead, under severe fluorination conditions, to highly fluorinated structures where the F/C ratio can approach 1. The bonds formed are then primarily stable covalent C–F bonds, reflecting high activation of the carbon network induced by the presence of electron-deficient sites. During the fluorination process, if B-containing functional groups are removed from the graphene lattice, a defect-rich graphene matrix can be formed, which enhances the F/C ratio and allows a high energy density of 2974 Wh/kg to be achieved [49].

The synthesis of boron and fluorine co-doped laser-induced graphene (FB-LIG) was achieved using a trifluoromethyl-functionalized precursor, enabling the scalable fabrication of high-performance micro-supercapacitors. Direct laser pyrolysis generates a porous three-dimensional graphene network with integrated heteroatoms that enhance defect density and surface wettability. This architecture optimizes ion diffusion and electrolyte accessibility while inducing charge polarization to accelerate transfer kinetics. Consequently, FB-LIG achieves a 23-fold increase in areal capacitance compared to pristine graphene. This dual-doping strategy efficiently engineers the electronic and structural properties of carbon, confirming superior stability and energy density for advanced electrochemical storage [64].

Colin et al. suggested a synergistic fluorination approach applied to sulfur-doped graphene (S-graphene) to develop efficient cathode materials for primary lithium batteries [65]. Controlled fluorination resulted in the coexistence of C–F and C–S bonds within the carbon structure and drove a partial transition from sp^2^ to sp^3^ hybridization. This structural evolution extended the interlayer spacing and reduced layer aggregation, producing a more open and porous morphology that improved electrolyte penetration and lithium-ion transport. In addition, the material exhibited higher discharge capacity, improved energy density, and more stable voltage behavior, highlighting the effectiveness of dual-doping strategies in optimizing graphene-based cathodes for advanced energy storage applications.

The synthesis of fluorinated N, P co-doped biomass carbon marks a substantial enhancement for Li/CFx batteries. The use of melamine and phytic acid as precursors leads to the formation of a defect-rich framework with an elevated F/C ratio and enhanced electrochemically active sites. This structural reconstruction stimulates the formation of semi-ionic C–F bonds, which compromise better accessibility and faster Li^+^ diffusion than traditional covalent bonds. The cathode exhibits high capacity and strong rate performance, controlled by greater ion transport and developed charge transfer within the synergistically doped carbon network [66].

#### 2.2.5. Curvature

**Figure 5:**
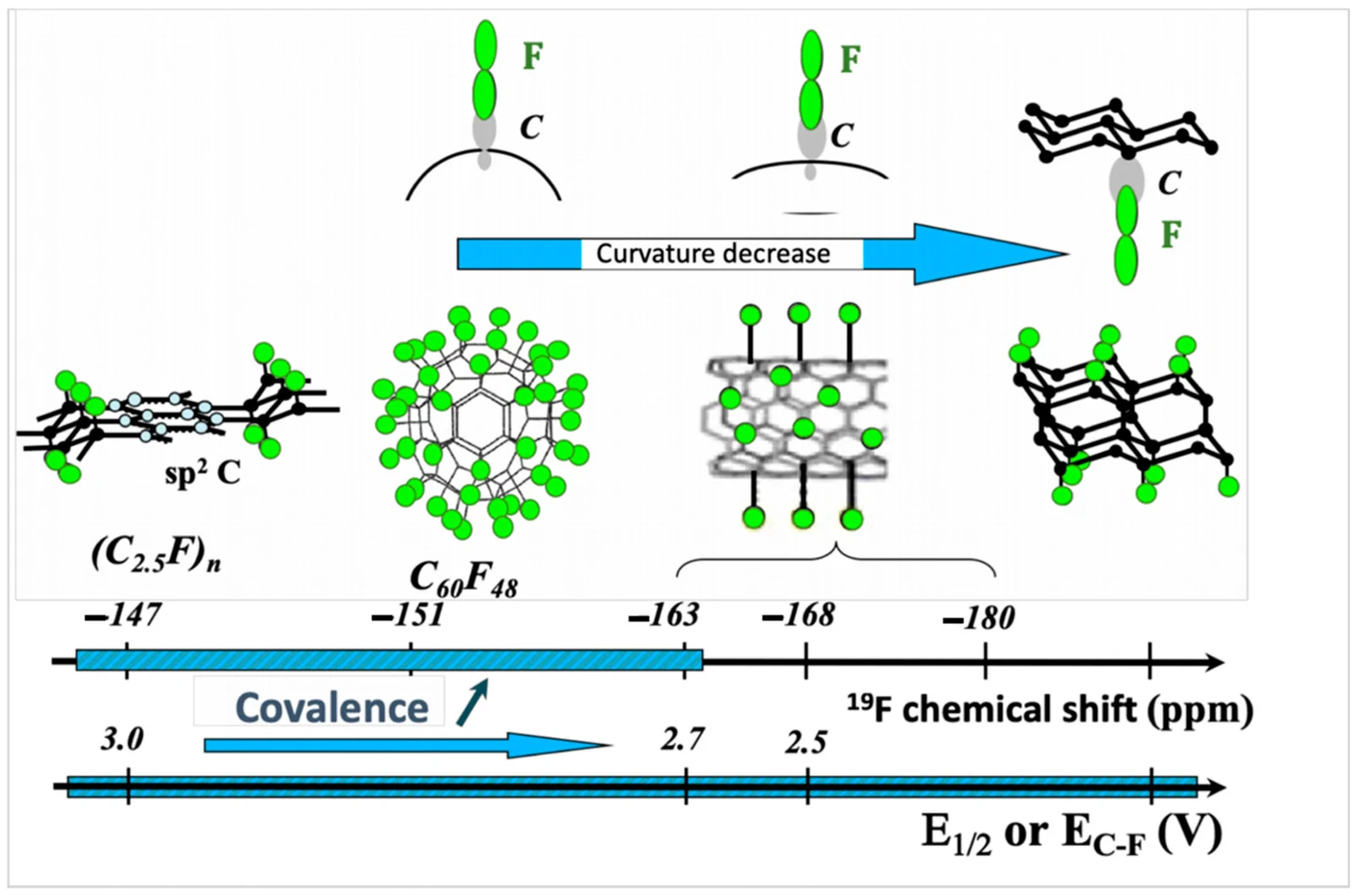
Effect of carbon framework curvature on C–F bond covalence, ^19^F NMR chemical shifts, and discharge potential (E12or EC − F) in primary lithium batteries across various fluorinated carbon nanostructures.

Curvature (ripples, topological defects) acts as an intrinsic structural activator of fluorination. DFT calculations revealed that curvature structure regulated its C–F bond configuration, thermodynamic parameters, and ion diffusion pathway [23]. In planar graphene or graphite, π electrons are delocalized and relatively stable, which leads to higher activation barriers for F_2_ adsorption and dissociation. In contrast, curved regions such as ripples, folds, nanotube-like geometries, or topological defects induce a partial rehybridization toward sp^3^ character and enhance the localization of π electron density. This electronic distortion weakens adjacent C–C bonds and increases their chemical reactivity toward electrophilic fluorine species. As a consequence, curved sites act as preferential nucleation centers for fluorination, where the initial activation of F_2_ is facilitated, and the formation of C–F bonds is energetically favored. Once initiated, the reaction can propagate from these highly reactive regions to less strained areas of the lattice [67,68]. With microspheres, the reduced size, narrower particle size distribution, and introduction of defects contribute to increasing the F/C molar ratio of F to C while preserving the electrochemical activity of C–F bonds [69]. Curvature hinders the effective overlap between the atomic orbitals of fluorine and carbon, resulting in a weakened C–F bond compared to a perfectly planar configuration. This reduction in bond strength is also evidenced by NMR chemical shifts (Figure 5), which reflect the altered electronic environment associated with curved structures. In a practical electrochemical context, this weakening of the C–F bond translates into a higher discharge potential when fluorinated carbon is used as the cathode material in lithium primary batteries, due to the reduced energy required for bond cleavage during electrochemical reduction.

### 2.3. Fluorination Products and Applications

2D fluorinated carbon materials exhibit properties that are highly dependent on the degree of fluorination and the sp^2^/sp^3^ structural organization, allowing for the distinction of three main regimes: partially fluorinated graphene (CFx), stoichiometric fluorographene (CF_n_), and bilayer F-diamane. Each of these states corresponds to a different trade-off s between conductivity, chemical stability, and mechanical rigidity, which directly determines their fields of application [16,70,71]. Partially fluorinated graphene (CFx, F/C ≈ 0.1–0.6) retains a significant fraction of sp^2^ lattice, which gives it a partial electrical conductivity ranging from ~10^−2^ to 10^2^ S/cm, depending on the connectivity of the residual graphitic domains. The simultaneous presence of covalent and semi-ionic C–F bonds, as well as CF_2_/CF_3_ terminal groups at the edges, induces significant electronic heterogeneity. The electronic bandgap is tunable between 0.1 and 1.5 eV, depending on the fluorination level and the spatial distribution of fluorine [72]. These properties make this material particularly suitable for chemical and biological sensors, where local charge variations enable the detection of gases such as NH_3_ [73] or NO_2_ [74] and biomolecules through conductivity modulation [33]. Graphene oxyfluoride membranes retained water vapor permeability similar to graphene oxide (8.7–9.1 LMH/bar) but with stable morphology, maintaining their densely packed structure and parallel arrangement of fluorinated sheets, highlighting their potential for tunable and stable separation applications [7]. It is also used in flexible electronic devices, notably field-effect transistors (FETs) whose performance can be adjusted by controlling the bandgap [75,76]. Enhanced light-matter interaction allows F-Gn to be used in a field-effect phototransistor [7]. The fluorescence emission of porous graphene with a high defect density exhibits a giant enhancement by controlled fluorination [77]. Finally, its hybrid structure makes it an excellent candidate for energy storage electrodes, with capacities in the range of 200–400 F/g for supercapacitors and 500–700 mAh/g for certain Li-ion battery architectures. Fluorographene (CF_n_, F/C ≈ 1) corresponds to a nearly complete conversion of the sp^2^ lattice to sp^3^, resulting in a material entirely saturated with strong covalent C–F bonds (≈485 kJ/mol). This structure confers the behavior of an electrical insulator with a very wide bandgap (≈3–4 eV) and very high resistivity (>10^12^ Ω·cm). Its surface is highly hydrophobic, with contact angles exceeding 120°, and the material exhibits marked chemical inertness [20,30,38,78]. Due to its high mechanical resistance and transparency, fluorinated graphene has been investigated as a coating for optical devices such as windows or lenses. Adhesion was improved through fluorination of graphene transferred onto a quartz substrate (a-SiO_2_) [79]. These properties open up several major applications: in energy, it is used as a cathode in lithium/fluorocarbon (Li/CF_x_) batteries, offering theoretical energy densities of up to 2180 Wh/kg with discharge voltages of 2.5 to 3.0 V [80]. Residual sp^2^ carbons (sub-fluorination) ensure the electron flux during the electrochemical process and enhance the power density (9828 W/Kg) [81]. Sub-fluorinated carbon nanofibers (F-CNFs) can be described as a non-fluorinated core surrounded by a fluorocarbon lattice. The core ensures electron flux in the cathode during electrochemical discharge in the primary lithium battery, which allows a high power density to be reached. The ball-milling in an inert gas (Ar) of these F-CNFs adds a second level of conductive sp^2^ carbons, i.e., a dual sub-fluorination. The structure changes from an initially similar multi-walled carbon nanotube to small lamellar nanoparticles after milling. The power densities are improved by the dual sub-fluorination [82]. A gradient fluorine content in the CFx powders resulted from high-density hydrogen plasma processing increased the ionic C–F bond amount on the surface of CFx. As a result, both the specific capacity and rate performance of the Li/CFx primary battery were significantly improved due to the enhanced electron and Li^+^ transfer evidenced by the electrochemical tests [83]. Recycled graphite from Li-ion batteries can be used as starting materials; the intrinsic defects in the precursor serve as active sites, promoting F_2_ reactions and lowering the C–F bond energy barrier, thereby enhancing fluorination thermodynamics [84]. Fluorination of boron-doped graphene removes B groups, creating a defect-rich graphene structure with more active sites and a higher F/C ratio. The resulting CFx delivers a high capacity of 1204 mAh·g^−1^ and an energy density of 2974 Wh·kg^−1^, outperforming non-doped and commercial fluorinated graphite. N, P co-doped biomass carbon (from melamine and phytic acid) was used as a precursor for CFx [85]. After fluorination, the material shows a high F/C ratio, a defect-rich porous structure, and removal of heteroatom groups, enhancing active sites and Li^+^ transport. It delivers 919 mAh·g^−1^ and 702 mAh·g^−1^ at 20C (76.4% retention), outperforming commercial fluorinated graphite and showing strong high-rate performance [66]. Oxifluorides offer the versatility to design cathode materials; The oxygenated regions contribute to durability, while the carbonaceous domains ensure electrical conductivity. An energy density of up to approximately 2400 Wh/kg is targeted [86]. Fluorinated microwave-exfoliated graphite oxide (MEGO) was produced by treating MEGO with XeF_2_ gas, introducing 5.7–29.3 at.% fluorine. XPS and IR analyses show that fluorine mainly binds to defective C–C sites while oxygen functional groups remain intact, slightly increasing the O/C ratio. Electrochemical tests reveal a 67% increase in specific capacitance, indicating improved double-layer performance and strong potential for supercapacitor applications [87]. Used as a cathode in fluoride-ion batteries (FIBs), graphite fluoride (CF)_n_ delivered 731 mAh.g^−1^ on discharge and 262 mAh.g^−1^ on charge (theoretical capacity of 864 mAh.g^−1^) whereas capacity of CuF_2_/Cu is 262 mAh g^−1^) [88]. During discharge, CF_n_ is electrochemically defluorinated into graphite-like carbon with intermediate (C_2_F)_n_ and (C_4_F)_n_ phases. During charge, fluorination mainly occurs at carbon edges, forming > CF_2_ groups, showing largely irreversible C–F bond breaking. The reversible electrochemical insertion/extraction of F^−^ ions occurs in carbon materials using graphite fluoride and a high-voltage Li-based electrolyte. The first discharge forms amorphous carbon and LiF with ~600 mAh·g^−1^ capacity (close to theoretical). The system shows ~200 mAh·g^−1^ reversible capacity with ~86% Coulombic efficiency and ~85% retention over 30 cycles, driven by reversible F^−^ transport linked to LiF formation [89].

**Figure 6:**
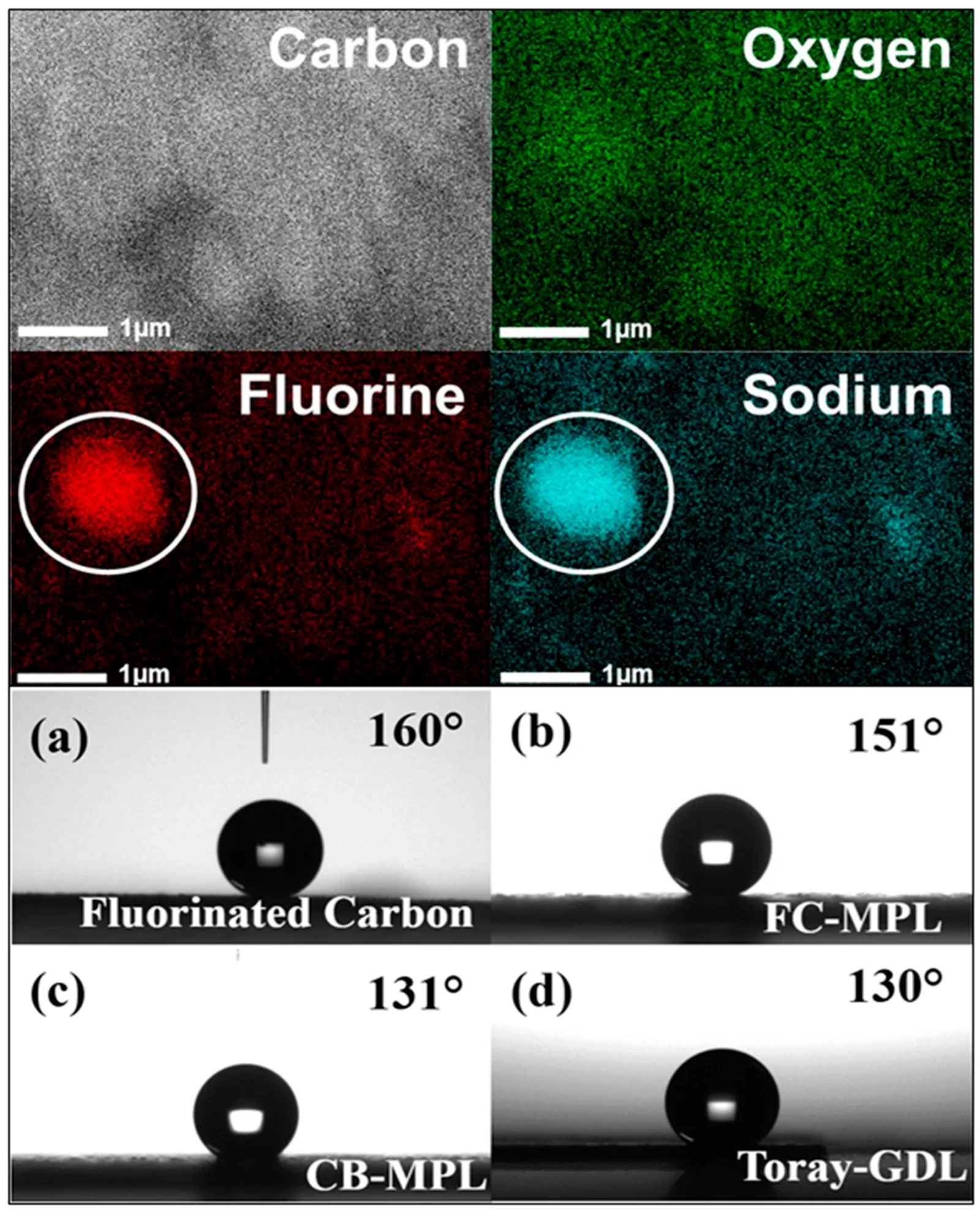
EDS elemental mapping and wettability of fluorinated carbon (FC) and related carbon substrates. EDS maps show the distribution of C, O, F, and Na. Contact-angle measurements for (**a**) fluorinated carbon (FC, 160°); (**b**) FC-MPL (151°); (**c**) CB-MPL (131°); and (**d**) Toray-GDL (130°), demonstrating their hydrophobic/superhydrophobic surfaces [76]. Adapted with permission from [90].

Can et al. produced fluorinated carbon nanoparticles using a solvothermal route and integrated them into microporous layers (MPLs) for polymer electrolyte fuel cells to improve water management (Figure 6) [90]. Fluorination introduced C–F groups, inducing partial sp^2^ to sp^3^ rehybridization while preserving the carbon framework, porosity, and conductivity needed for efficient gas transport. The high electronegativity of fluorine lowered surface energy, increasing hydrophobicity and promoting higher water contact angles, which helped prevent water accumulation and electrode flooding. Consequently, the modified MPLs showed reduced mass transport resistance, improved oxygen diffusion under humid conditions, and enhanced current–voltage performance compared to traditional carbon/PTFE-based systems.

**Figure 7:**
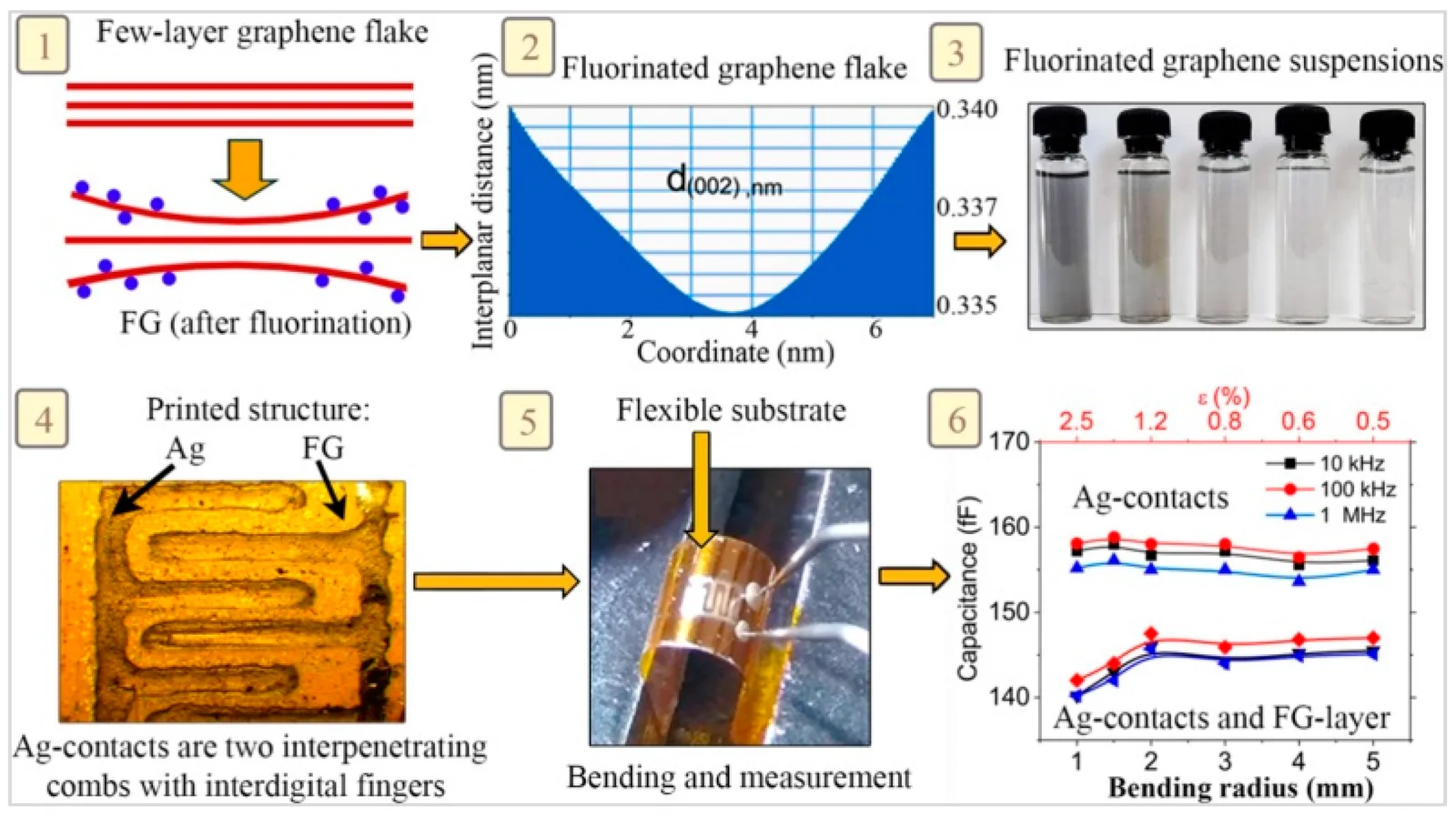
Schematic illustration of the preparation and characterization of fluorinated graphene (FG): (1) few-layer graphene flake before and after fluorination, where blue circles represent fluorine atoms; (2) fluorinated graphene flake; (3) FG suspensions; (4) printed FG structure with Ag contacts; (5) FG on a flexible substrate during bending measurements; and (6) capacitance as a function of bending radius [91]. Adapted with permission from [91].

Fluorination of graphene has been widely explored for flexible and printed electronics (Figure 7) [91]. Commonly, the process starts with liquid-phase exfoliation of bulk graphite to obtain few- or single-layer graphene flakes. This step is essential because it increases the available surface area and exposes reactive sites, allowing fluorine to be incorporated more uniformly. Controlled fluorination then leads to the formation of C–F bonds with adjustable coverage. As a result, the graphene structure is partially disturbed, shifting from an sp^2^ to a more sp^3^-like composition, which presents defects and slightly expands the interlayer spacing. These changes improve dispersibility and serve stable ink generation for thin films. In parallel, fluorination modifies the electronic behavior, gradually turning graphene from highly conductive into semiconducting or insulating, based on fluorine content. Electrochemically, this balance boosts sensitivity and selectivity, generating functional graphene via fluorination useful in sensing and electronic applications.

Carbon-based nanomaterials are widely used as solid lubricant additives because of their excellent wear resistance and their low coefficient of friction (COF), typically ranging from 0.05 to 0.10 when dispersed in suitable lubricating media. Covalent grafting of fluorine atoms can further improve their tribological performance, leading to COF values as low as 0.03–0.10 depending on both the structure and dimensionality of the fluorinated nanomaterial [92,93,94]. The optimum fluorination rate required to achieve the lowest coefficient of friction depends strongly on the dimensionality of the carbon nanomaterial. The relationship between fluorination degree, nanocarbon dimensionality, and tribological performance has recently been comprehensively reviewed [28]. During tribological testing, 2D materials tend to form a tribofilm at the sliding interface, which can influence friction and wear behavior. Fluorinated 2D materials, such as fluorographene and partially fluorinated graphene (C × F), maintain low friction coefficients owing to their reduced surface energy, typically in the range of 20–30 mJ m^−2^. This low surface energy facilitates interlayer sliding and limits adhesive interactions between contacting surfaces. As a result, fluorinated graphene-based materials exhibit enhanced lubricating properties and sustain reduced friction even in the presence of tribofilm formation. Further decreases in COF can be obtained through their use as additives to already lubricating oils or organic solvents [28]. CF× exhibit COF similar to that of PTFE but with superior thermal stability as they can withstand temperatures as high as 500 °C without sustaining degradation [95]. Other properties can be influenced by introducing metal additives into the tribofilms, such as LiF or AlF_3_, acting to protect metal surfaces from additional wear [96], compared to traditional lubricants (MoS_2_), a 50–80% reduction in wear.

## 3. Fluorination of Hexagonal Boron Nitride (H-BN)

**Figure 8:**
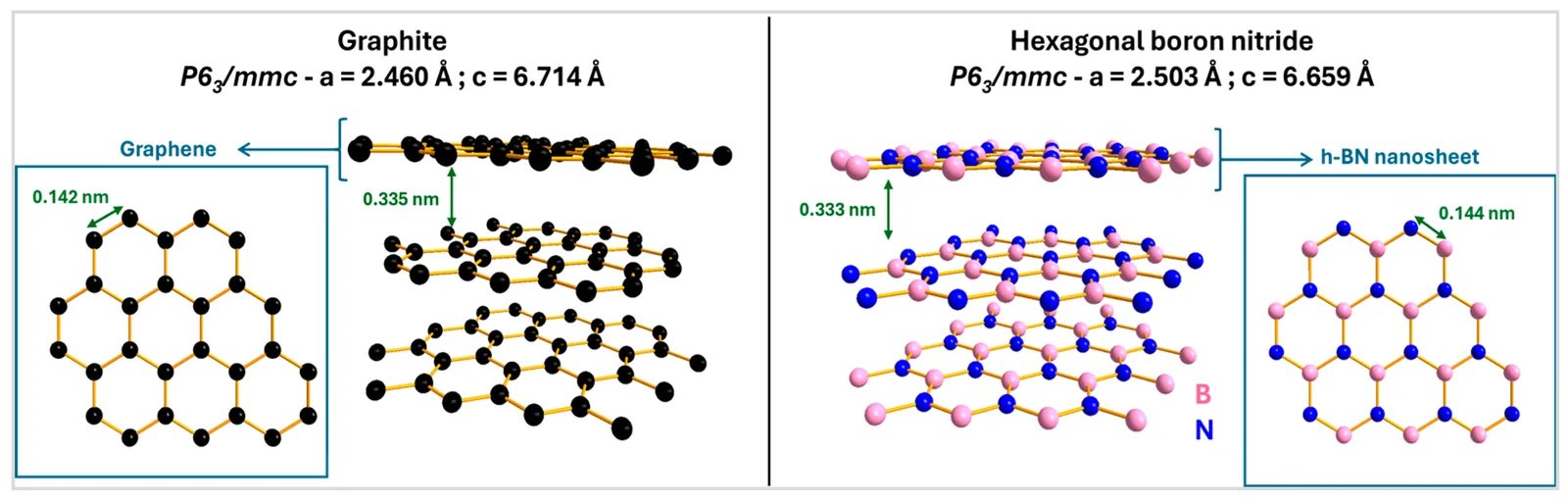
Structural comparison between graphite and h-B-N, highlighting their space group symmetry $\left(P6_{3}/mmc\right)$, lattice parameters, interlayer spacing, and in-plane bond lengths.

h-BN is a 2D layered material composed of alternating boron and nitrogen atoms arranged in a graphene-like honeycomb lattice (Figure 8) [97,98,99,100]. Often referred to as “white graphene”, it displays exceptional thermal stability, chemical inertness, and high mechanical strength. In contrast to graphene, h-BN is an electrical insulator with a wide band gap, which limits its direct use in electronic and electrochemical devices. However, this property makes it highly valuable as a dielectric, thermal management medium, and protective coating. Its layered structure enables efficient exfoliation into ultrathin nanosheets with high surface area and mechanical flexibility. Moreover, its chemically inert nature and structural robustness provide a versatile and cost-effective platform for post-synthetic functionalization, enabling precise tuning of surface chemistry, morphology, and electronic properties for advanced applications.

### 3.1. Gas-Solid Fluorination of H-BN

Although h-BN and graphene have a similar structure (Figure 8), h-BN is a much more reactive material. Most direct fluorinations of h-BN with F_2_ were carried out at room temperature or at moderate temperatures (below 100 °C), unlike graphene. Furthermore, direct fluorination of h-BN tends to destroy the material rather than perform covalent grafting, forming gaseous species such as BF_3_, NF_3_, or N_2_. This necessitates carrying out fluorination under diluted conditions with an inert gas such as helium or nitrogen. Meiyazhagan et al. performed controlled fluorination of h-BN by direct fluorination with highly diluted fluorine (10% in He) at room temperature and at 50 °C [101]. The use of N_2_ as a diluent is also possible, as demonstrated by Amarathunga et al., where h-BN and BN nanotubes (BNNTs) were fluorinated with 30% dilute F_2_ at temperatures between 15 and 50 °C [102]. Whether h-BN or BN nanotubes are involved, the reaction mechanism is the same: fluorination occurs preferentially at boron atoms, creating B–F bonds identifiable by solid-state NMR, infrared spectroscopy, and XPS. DFT calculations confirm this preference of fluorine for the boron atom, although N–F bonds are also quite possible, as observed experimentally. These modifications improved the electronic structure, enhanced interfacial electron transfer, and increased surface reactivity. Consequently, fluorinated BN (h-BN or BNNTs) displayed improved electron transfer kinetics, lower charge transfer resistance, and enhanced electroanalytical performance, underlining their potential for sensing, catalysis, and energy-related applications. Fluorinated h-BN (F-hBN) was synthesized through a scalable, post-exfoliation gas-phase fluorination sequence that confirms homogeneous fluorine incorporation while maintaining the structural integrity of the fluorinated materials [103,104]. This chemical modification stimulates critical physicochemical shifts, including interlayer lattice expansion, amplified surface polarity, and improved dispersibility, while maintaining the fundamental crystalline framework of the h-BN precursor. Salpekar et al. report that h-BN fluorinated with diluted F_2_ reveals a marked enhancement in thermal conductivity, while simultaneously displaying a significant reduction in friction and wear of about 31% and 71%, respectively [17]. Furthermore, the induction of permanent dipole moments enhances the dielectric response of the material. Consequently, targeted fluorination serves as a refined engineering strategy to control the electronic and interfacial characteristics of h-BN, expanding its function across high-performance thermofluids, progressive lubricants, and next-generation microelectronic constructions.

Schematic illustration of h-BN highlighting the similarities with graphite.

The use of solid fluorinating agents is also proposed to tune the properties of h-BN. Xenon difluoride (XeF_2_) was used by Shin et al. to perform the etching of h-BN at room temperature in the presence of a silicon substrate [4]. The first step consists of the activation of XeF_2_ by the silicon surface (denoted as Si*), thus forming the XeF intermediate species (Equation (3)):

(3) $$Si\;-\;{Si}^{*}\;+\;XeF_{2}\;\to \;{\left[Si\;-\;F.Si\right]}^{*}\;+\;XeF$$

Further fluorination of the silicon surface ultimately yields SiF_4_. XeF then reacts with h-BN to yield BF_3_ and N_2_ according to the following reaction (Equation (4)):

(4) $$2BN\;+\;6XeF\;\to \;2{BF}_{3}\;+\;\;N_{2}\;+\;6Xe$$

XeF_2_ both functionalizes and cleaves B–N bonds, producing BF_3_ and N_2_ with electron release, leading to thinning, defects, and structural reconstruction of h-BN. XeF_2_ can still be an effective fluorinating agent for h-BN, with covalent grafting of F atoms onto boron and nitrogen, as well as intercalation of fluoride ions in the interlayer space as demonstrated by Takami et al., thus making the material an excellent conductor of fluoride ions for all-solid-state fluoride-ion battery applications [105].

A final gas-solid reaction fluorination method for h-BN involves the use of fluorinated plasmas. This method is less aggressive to the material and allows for precise tuning of the fluorination level by controlling the amount of plasma gas and the power of the RF generator. Zhong et al. successfully prepared BN nanosheets by ball-milling from h-BN, then fluorinated them with an RF-induced Ar/CF_4_ plasma [106]. The fluorinated materials are then incorporated into a glass fiber reinforced polymer composite, which offers enhanced electrical insulation properties compared to the pristine material.

### 3.2. Fluorination of H-BN via Wet Methods

The complexity of using molecular fluorine has led many authors to prefer alternative fluorination pathways that do not involve F_2_. NH_4_F is a widely used fluorinating agent because it is readily available. Pure NH_4_F_(s)_ has a fairly low decomposition temperature, yielding NH_3(g)_ and HF_(g)_ as the main products at approximately 100 °C. However, most authors prefer using it in aqueous solutions in which the F^−^ anion is the fluorinating agent. Du et al. performed a one-step exfoliation and fluorination of h-BN by a hydrothermal method involving NH_4_F as fluorinating agent in aqueous media [107]. While F^−^ ions chemically bind to the boron atoms, NH_4_^+^ assists the exfoliation process by intercalating itself between the h-BN sheets. Such materials exhibit interesting magnetic properties that does not exist in the bulk. Hydrothermal synthesis with NH_4_F can also be combined with mechanical activation of materials via ball-milling. Ul Ahmad et al. successfully activated h-BN by mechanical milling followed by hydrothermal treatment with NH_4_F [103], while Ding et al. propose performing the hydrothermal treatment first, then performing the ball-milling process [104]. Mechanochemical fluorination of h-BN directly in an aqueous ammonium fluoride (NH_4_F) medium also offers an effective one-pot strategy for simultaneous exfoliation and surface functionalization [108]. High-energy ball milling introduced structural defects and lattice strain, thereby activating the otherwise chemically inert h-BN and facilitating fluoride incorporation primarily through B–F bond formation at boron-rich sites. The process partially disturbed the sp^2^ lattice and promoted the growth of scroll-like nanostructures. These structural modifications enhanced surface polarity and aqueous dispersibility, while the fluorinated nanosheets revealed enhanced tribological behavior by forming nanoscroll structures that acted as nano-ball bearings, reducing friction and wear. A recent study demonstrated that cryomilling of h-BN at the temperature of liquid nitrogen (−196 °C) followed by fluorination with NH_4_F in aqueous solution enabled high fluorine incorporation into h-BN through selective B–F bond formation while conserving biocompatibility [109]. The fluorination reduced the bandgap and introduced localized electronic states, adapting h-BN from an inert insulator into an electrochemically active electron-accepting material. The modified h-BN also exhibited enhanced interfacial charge transfer and strong antibacterial activity, achieving 99% efficiency against *E. coli* through disruption of bacterial electron transport and metabolic balance. These findings highlighted fluorinated h-BN as a promising platform for antimicrobial and biosensing applications. Lastly, fluorination of ball-milled activated BN nanosheets followed by hydrothermal treatment with NH_4_F also offers interesting applications in Surface-enhanced Raman spectroscopy (SERS), which, in combination with gold nanoparticles, can significantly increase the Raman scattering of molecules [103].

Among other wet fluorinating agents, tetrafluoroboric acid (HBF_4_) in solution can also be used as a fluorine source. Venkateswarlu et al. have performed fluorination of h-BN nanosheets via a solution-based F-doping approach using HBF_4_ as the fluorine source [110]. This chemical functionalization is made highly effective by the previous exfoliation of bulk h-BN in an alcoholic medium; this morphological shift into few-layer 2D nanosheets greatly increases the surface area and provides numerous reactive edge sites, confirming a homogeneous incorporation of fluorine. This process disrupts the pristine sp^2^ hybridized network and introduces controlled defects, which narrows the band gap and shifts electronic nature from a wide-band-gap insulator toward a defect-engineered semiconductor. When applied to magnesium-ion batteries, this electronic restructuring and increased surface roughness enhance charge redistribution and improve Mg^2+^ adsorption. Consequently, these modifications accelerate charge-transfer kinetics at the electrode–electrolyte interface, enabling h-BN flakes to deliver a reversible magnesium-ion storage capacity of 50 mAh g^−1^. This demonstrates that targeted structural engineering via exfoliation and fluorination can efficiently activate layered insulators as functional cathode materials for advanced energy storage systems.

The fluoropolymer Nafion can also be used as a fluorinating agent, using solvothermal methods in organic solvents such as dimethylformamide (DMF). Radhakrishnan et al. reported the fluorination of h-BN with Nafion as an effective way to modify its electronic and magnetic properties [111]. In this study, fluorine atoms are covalently integrated into the h-BN lattice, specifically bonding with boron sites to form B–F configurations, which induce localized structural distortions and disrupt the intrinsic symmetry of the sp^2^-bonded network. Unlike conventional strict functionalization attitudes, fluorination introduces controlled defect states without completely breaking the crystallinity of h-BN, thereby preserving its layered morphology while significantly modifying its physicochemical performance. The incorporation of highly electronegative fluorine induces charge redistribution and localized magnetic moments, transforming insulating h-BN into a spin-polarized magnetic semiconductor with a reduced band gap. Fluorination induces slight lattice distortion and increased interlayer spacing, while introducing mid-gap states that facilitate charge transport and magnetic ordering, thereby expanding h-BN applications to spintronics, magnetic sensing, and quantum electronics. Fan et al. also report successful fluorination by Nafion in DMF of mechanically activated h-BN nanosheets, which exhibits promising tribological behaviors [112].

**Figure 9:**
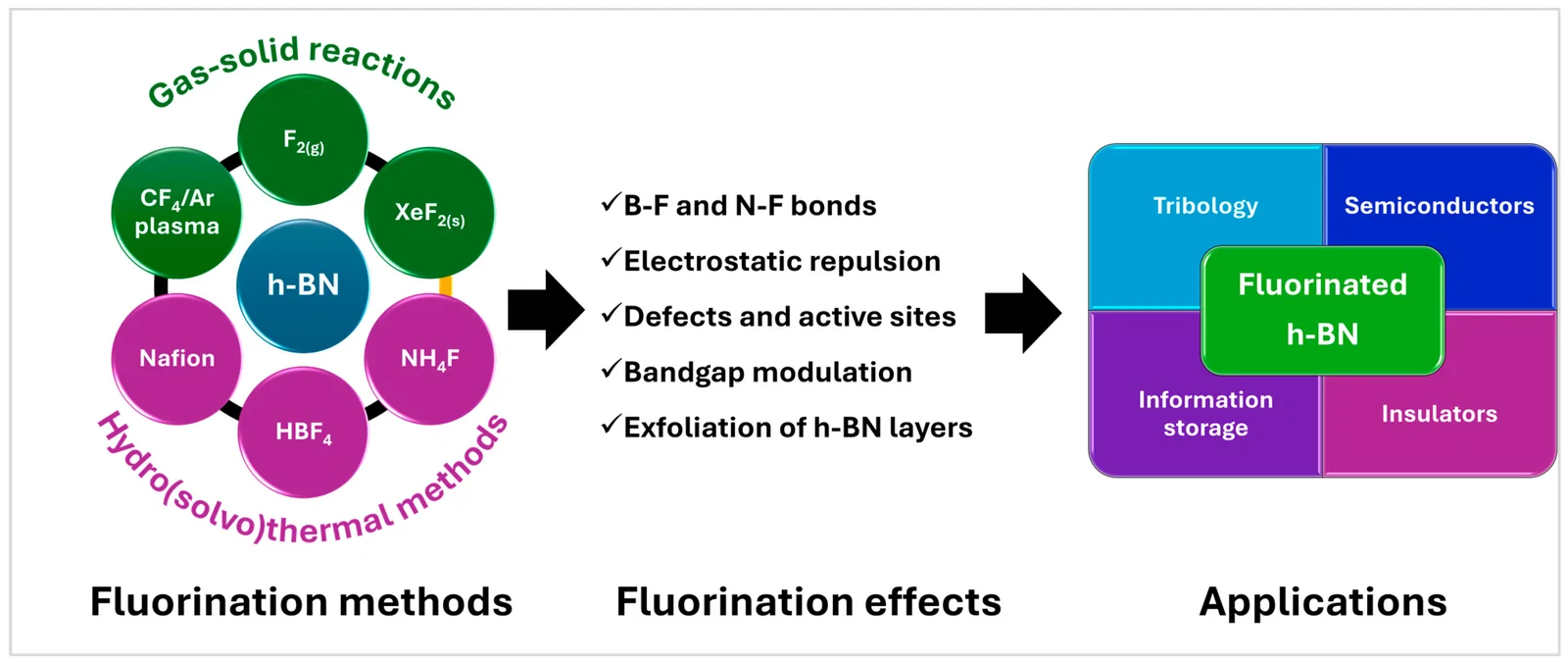
Synthetic routes (gas-solid and hydro/solvothermal), induced structural/electronic effects, and key applications of fluorinated h-BN.

Controlled fluorination of h-BN, whether with molecular fluorine F_2_ or via the use of fluorinating agents in liquid medium, gives the material new properties through the incorporation of a highly electronegative element. Among the targeted applications, bandgap modulation offers the possibility of use in semiconductors or catalysis. The electrostatic repulsion between fluorine atoms can find applications in tribology, similar to fluorinated carbons already used as solid lubricants. The incorporation of fluorinated h-BN into composites can also provide these materials with increased resistance to high temperatures due to the high intrinsic thermal conductivity of h-BN. Finally, fluorination of h-BN nanosheets confers magnetic properties that did not exist in the pristine material, making them suitable for use in spintronics for information storage (Figure 9).

## 4. Fluorination of G-C_3_N_4_

Graphitic carbon nitride (g-C_3_N_4_) is a layered π-conjugated inorganic polymer composed of graphene-like sheets. Its structure is based on nitrogen-linked aromatic units that can be either triazine (C_3_N_3_) or heptazine (C_6_N_7_) motifs [113]. Since heptazine units are thermodynamically more stable, they are generally considered the fundamental building blocks of g-C_3_N_4_. The individual layers are connected through weak van der Waals interactions, resulting in a lamellar structure. However, its layered architecture results in a relatively low specific surface area, typically around 10 m^2^ g^−1^, thereby limiting its photocatalytic performance [114]. The stacking arrangement of these layers remains under debate and may adopt different configurations, depending on the relative positions of adjacent layers. Accurate determination of the crystal structure is challenging because g-C_3_N_4_ synthesized by polycondensation typically exhibits low crystallinity and limited structural order. Consequently, structural descriptions of g-C_3_N_4_ generally assume a framework exclusively composed of heptazine units, regardless of the exact stacking sequence of the layers. Graphitic carbon nitride (g-C_3_N_4_) is a visible-light-responsive n-type semiconductor that has attracted considerable attention for photocatalytic applications, particularly in the degradation of organic pollutants in aqueous media. Its indirect bandgap of approximately 2.7 eV enables efficient absorption of visible light, while its band-edge positions are thermodynamically suitable for water oxidation, oxygen reduction to superoxide radicals, and hydrogen evolution [113].

Among the various heteroatom-modification strategies investigated for graphitic carbon nitride, fluorination remains relatively underexplored, with only around twenty studies reported to date. Most fluorination procedures rely on solvothermal treatments using ammonium fluoride, hydrofluoric acid, or related fluorinated precursors [115,116,117,118,119,120]. These approaches generally result in low fluorine incorporation levels, typically below 1 at.%. Nevertheless, even such limited fluorine contents have been shown to significantly modify the structural, electronic, and photocatalytic properties of g-C_3_N_4_, consistently leading to enhanced photocatalytic performance compared with the pristine material.

Gas–solid fluorination processes, where fluorine is supplied by a gaseous precursor, have received very limited attention in the literature. The use of molecular fluorine (F_2_) as a fluorinating agent has been reported only twice. Sun et al. achieved fluorine concentrations as high as 7 at.% by treating g-C_3_N_4_ with a 20 vol.% F_2_/N_2_ gas mixture at temperatures ranging from 120 to 180 °C [121]. This relatively high fluorine loading resulted in a substantial increase in specific surface area, from 11.5 to 74 m^2^ g^−1^, accompanied by improved charge separation and a slight reduction in photoluminescence intensity. Buzdon et al. have highly fluorinated g-C_3_N_4_-F (~19 at.% F) via direct F_2_/N_2_ gas fluorination at room temperature [122]. The fluorination induces structural defects and nitrogen vacancies, exfoliation, and the formation of covalent C–F bonds. Fluorination markedly enhanced the optoelectronic properties of g-C_3_N_4_, leading to increased photogenerated charge density, prolonged carrier lifetime, reduced recombination, and improved charge-transfer efficiency. Consequently, the photocatalytic degradation of methyl orange increased from 19.9% for pristine g-C_3_N_4_ to 92.8% for fluorinated g-C_3_N_4_-F, while carbamazepine degradation increased from 25% to 35% with different degradation products. The enhanced photochemical activity originated from the combined effects of structural and electronic changes due to fluorine incorporation, fluoride-ion release, and solution acidification, which promoted charge carrier photogeneration, pollutant adsorption, and reactive oxygen species generation.

The treatment with xenon difluoride promoted the formation of cyano functionalities and, to a lesser extent, nitrogen vacancies, resulting in bandgap narrowing from 3.06 to 2.86 eV and nearly complete suppression of radiative recombination pathways. These modifications dramatically enhanced photocatalytic hydrogen evolution, yielding H_2_ production rates approximately 14.5 times higher than those of pristine and fluorinated g-C_3_N_4_. The improved performance was attributed to increased charge-carrier density and the formation of additional electronic states near the conduction band. Interestingly, fluorination alone reduced the photoluminescence lifetime, whereas subsequent defluorination increased it significantly, highlighting the critical role of fluorine-induced defects in controlling charge dynamics [123]. The second XeF_2_-based study focused on fluorinated g-C_3_N_4_ quantum dots prepared through a photochemical route [123]. Despite fluorine contents reaching 10.56 at.%, the microscopic structure remained largely unaffected. Increasing fluorine incorporation was accompanied by a decrease in oxygen content, likely resulting from the oxidative decomposition of oxygen-containing surface groups by XeF_2_. The resulting modifications produced pronounced red shifts in photoluminescence emission and enhanced charge interactions. In addition, oxygenated groups were proposed to act as recombination centers; their replacement by fluorine reduced non-radiative recombination pathways and increased photoluminescence efficiency. A reduction in bandgap energy was also observed.

From a structural perspective, fluorination commonly induces a reduction in sheet sizes, pore formation, and a decrease in sheet thickness, resulting in fewer stacked layers [120,121,124]. However, the precise location of fluorine atoms within the g-C_3_N_4_ framework remains a matter of debate and strongly depends on both the fluorination method and precursor employed. Spectroscopic analyses suggest that fluorine predominantly substitutes nitrogen atoms, leading to the formation of C–F bonds. In particular, tri-coordinated bridging nitrogen atoms appear to be preferentially substituted due to their lower bond energies compared with ring nitrogen atoms. DFT calculations further indicate that fluorine preferentially adsorbs onto sp^3^ carbon sites and amino groups located at sheet edges, forming covalent C–F bonds [125]. The most favorable adsorption sites are carbon atoms bridging adjacent triazine units, which subsequently facilitate nitrogen substitution. As a consequence of fluorine’s high electronegativity, partial conversion of carbon from sp^2^ to sp^3^ hybridization occurs, inducing local structural disorder and generating dangling bonds. These modifications may introduce localized dipolar moments and defect states within the bandgap, thereby influencing charge-carrier mobility and electronic structure [23,111]. The nature of the C–F bond itself is strongly dependent on fluorination conditions, fluorine concentration, and synthesis methodology. Covalent, semi-ionic, and ionic C–F bonds have all been reported [40,126]. The nature of the C–F bond strongly influences the electronic conductivity of the material, thereby affecting charge-transport properties and charge-carrier separation efficiency. Consequently, controlling the bonding environment represents a key parameter for tailoring the functional properties of fluorinated g-C_3_N_4_. Several fluorination mechanisms have been proposed in the literature. Lin et al. suggested direct substitution of bridging nitrogen atoms by fluorine atoms [126]. Ma et al. proposed a fluorination-induced exfoliation mechanism involving initial fluorine attachment to surface carbon atoms, triazine ring opening, formation of F–C–N–H species, and progressive intercalation of fluoride and ammonium ions, ultimately leading to layer-by-layer exfoliation [118]. In contrast, Sun et al. proposed that fluorine atoms bond to carbon atoms adjacent to bridging nitrogen sites, weakening N–C_3_ bonds and facilitating the formation of volatile NF_3_ through reaction with fluorine radicals [121]. This process generates nitrogen vacancies and significant lattice distortions.

**Figure 10:**
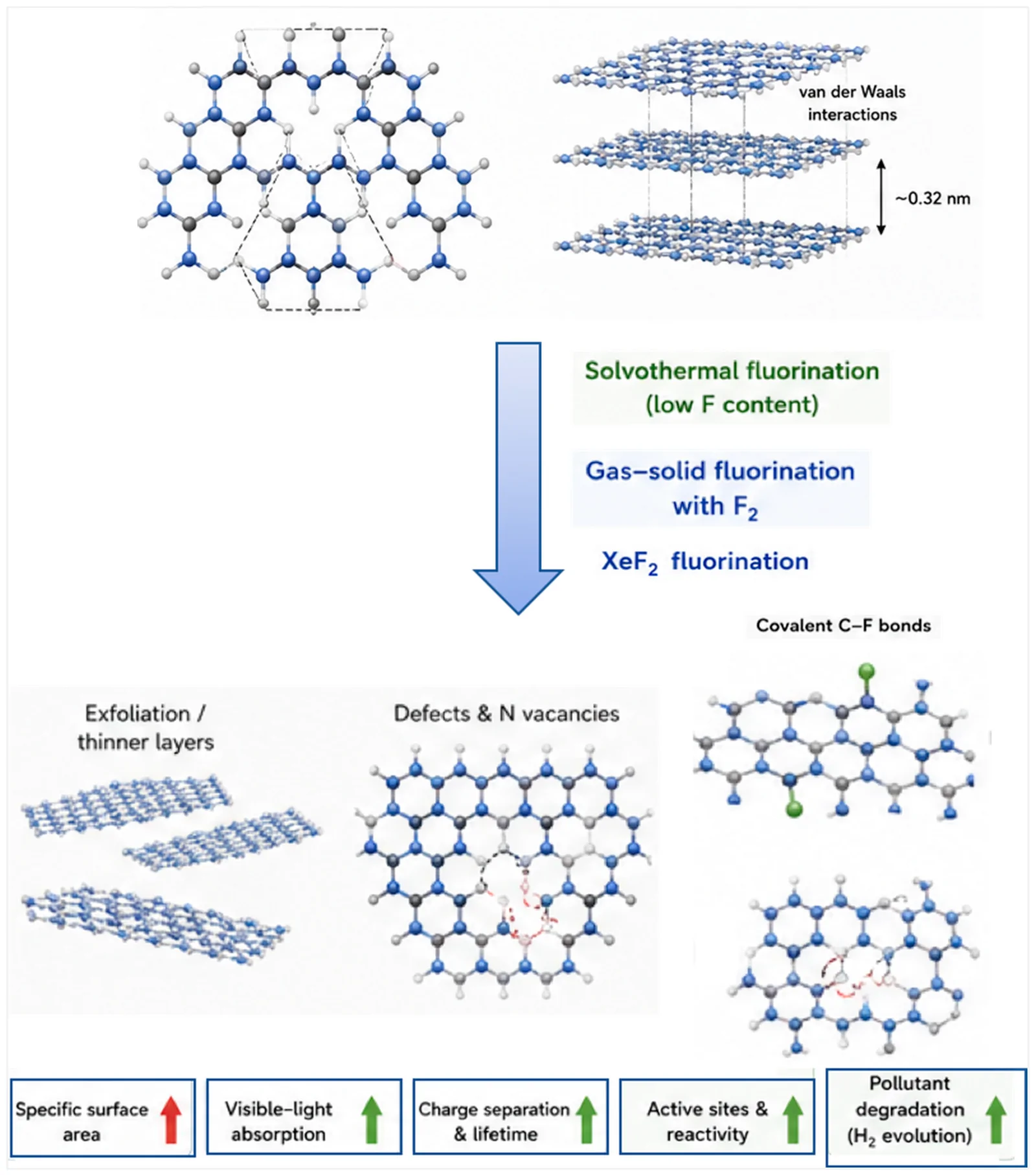
Schematic overview of the fluorination of graphitic carbon nitride (g-C_3_N_4_), illustrating the main fluorination routes and their effects on the structure, electronic properties, and photocatalytic performance.

Fluorination parameters exert a profound influence on the resulting material structure. Excessively harsh fluorination conditions may severely damage the g-C_3_N_4_ framework, leading to excessive fragmentation and the formation of energetic barriers that hinder charge transport and reduce photocatalytic efficiency [115]. Therefore, fluorination conditions must be carefully optimized according to the targeted application, balancing defect generation against structural preservation. Although fluorination frequently increases the specific surface area of g-C_3_N_4_, the opposite effect has also been reported in certain cases due to pore shrinkage or weaker gas-surface interactions [40]. Similarly, the influence of fluorination on the bandgap remains controversial. Depending on the fluorination route and fluorine content, both bandgap widening [118,121] and narrowing [115,116,121] have been reported. Bandgap enlargement has been attributed by Sun et al. to fluorination-induced structural distortion [121], whereas according to Alam et al., bandgap reduction may indicate effective incorporation of fluorine into the carbon nitride framework through covalent C–F bonding rather than fluorine radical surface adsorption through an ionic bond [127]. Importantly, bandgap narrowing extends the visible-light absorption range and is generally associated with enhanced photocatalytic activity [119]. Overall, fluorination improves photocatalytic performance through several synergistic effects, including enhanced visible-light absorption, accelerated charge transport, and suppressed electron-hole recombination resulting from modification of the π-conjugated electronic structure [128,129]. Nevertheless, the precise mechanisms underlying pollutant photodegradation remain poorly understood. By analogy with other halogens, fluorine may participate in the formation of reactive radical species capable of contributing to photocatalytic degradation pathways [130,131]. Figure 10 and Table 1 summarize the main recent works.

## 5. Fluorination of Other 2D Materials, MXene

**Figure 11:**
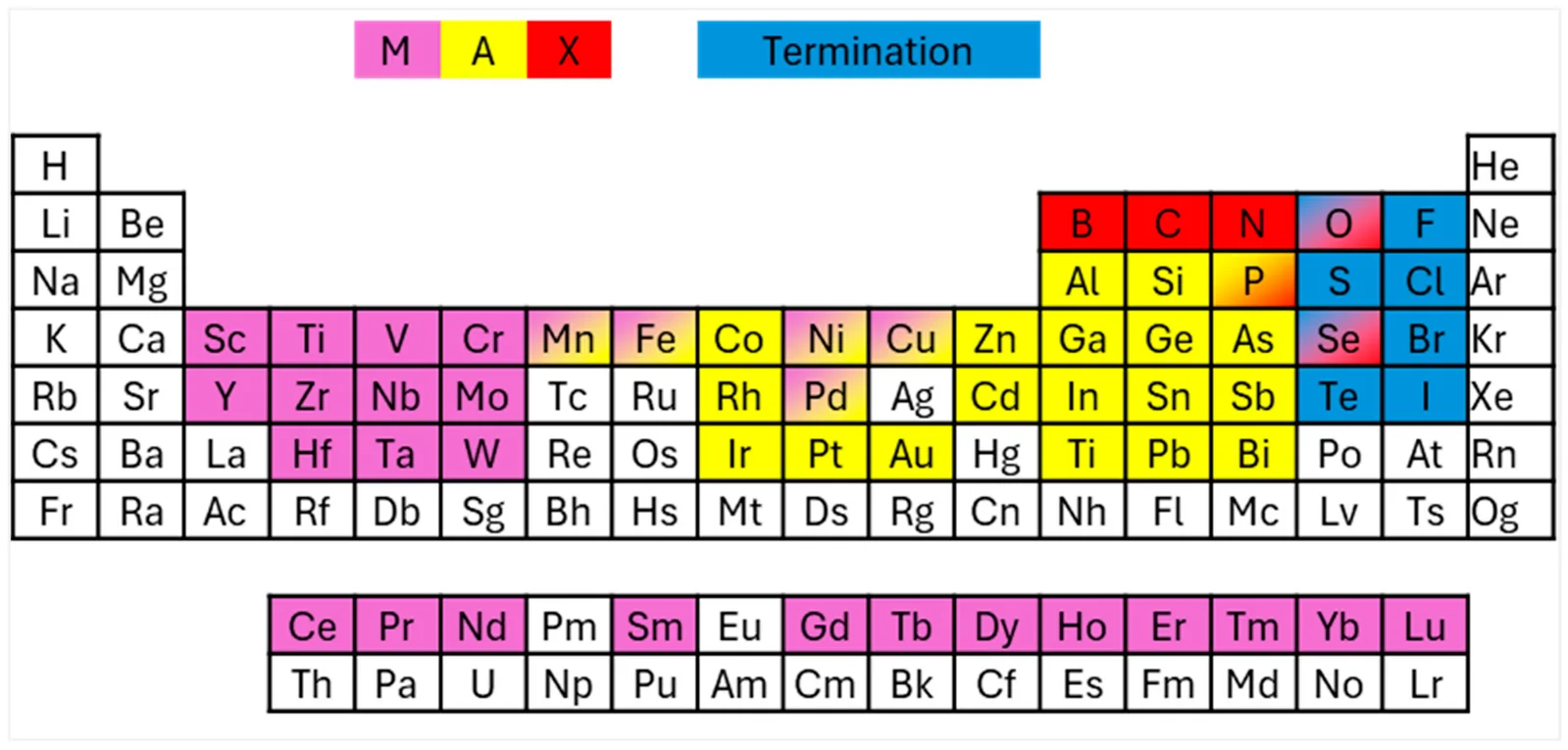
Elements present in the MAX phases and/or MXene synthesis.

MAX phases are layered structures presenting a general formula under the form M_n+1_AX_n_ (n = 1–6), in which M is an early transition metal or rare-earth elements, A is an A-group element, X is C, N, B, O, Se and/or P. The following figure presents the wide array of elements that can be found in the variety of MAX phases (Figure 11). Additional components have a high probability of being incorporated into these structures in the future.

**Figure 12:**
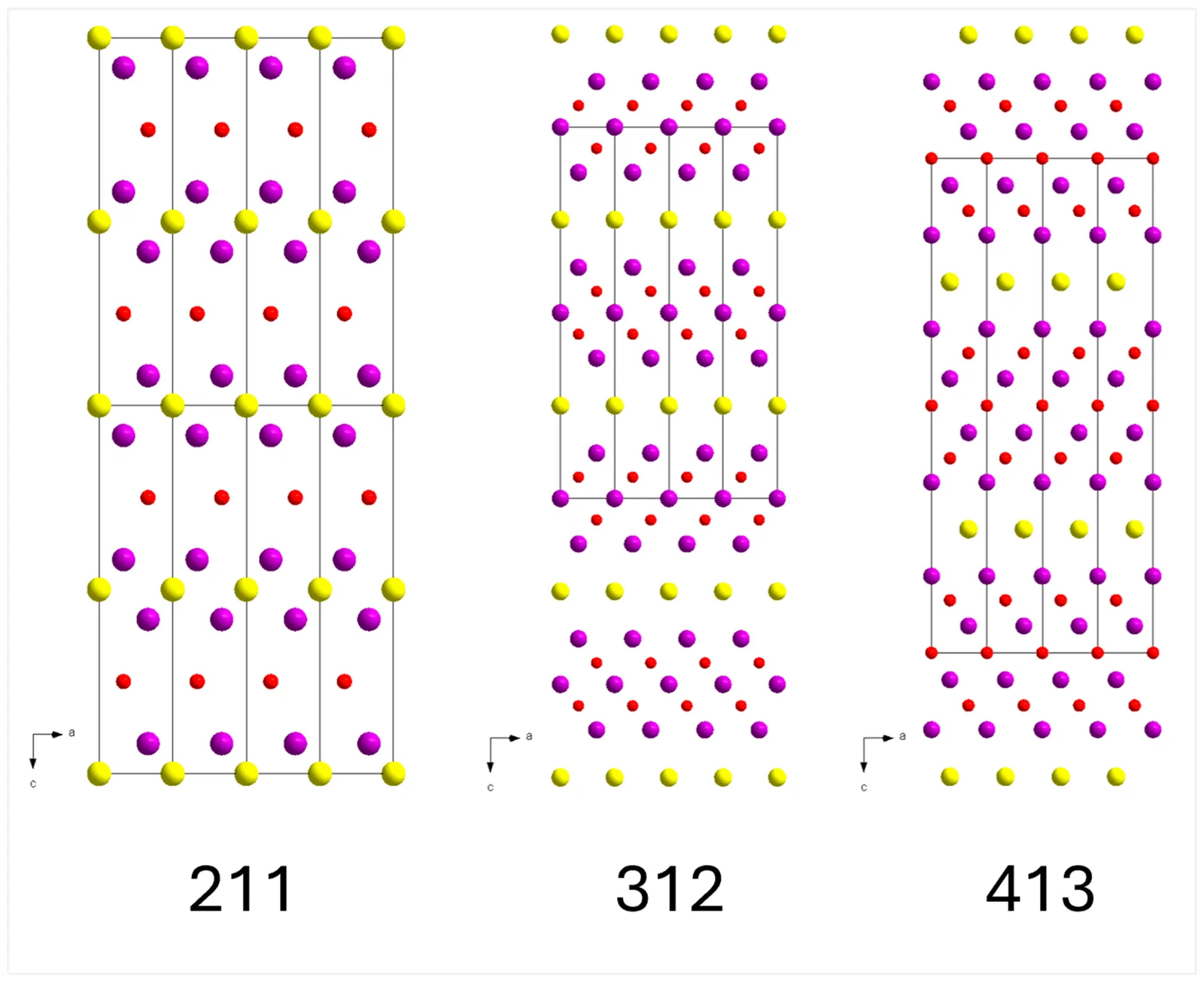
Crystal structures and atomic stacking sequences of MAX phases: 211 (n = 1), 312 (n = 2), and 413 (n = 3). The structures follow the general formula M_n+1_AX_n_, where M is a transition metal, A is a group 13 or 14 element, X is C and/or N, and n represents the number of M–X layers between A layers. Purple, yellow, and red spheres denote M, A, and X atoms, respectively, highlighting the increasing M–X slab thickness from 211 to 413.

MAX phases are classified by the value of n, resulting in subcategories such as M_2_AX (211 phase), M_3_AX_2_ (312 phase), M_4_AX_3_ (413 phase), M_5_AX_4_ (514 phase), M_6_AX_5_ (615 phase), and M_7_AX_6_ (716 phase). These phases present a stacking of metallic A layers separated by a carbide layer (211), 2 carbide layers (312), 2 carbide layers on either side of a graphite layer (413) and henceforth, following the value of n. Some MAX phases such as (Mo_2/3_Y_1/3_)_2_AlC, (Mo_2/3_Sc_1/3_)_2_AlC, (W_2/3_Sc_1/3_)_2_AlC, (W_2/3_Y_1/3_)_2_AlC, (V_2/3_Zr_1/3_)_2_AlC, display layers containing two transition metals, these are named iMAX phases (Figure 12) [143,144,145,146]. Among them, Ti_2_AlC and Ti_3_SiC_2_ are the most extensively studied and have been used most frequently to synthesize MXenes. Among them, Ti_2_AlC and Ti_3_SiC_2_ are the most extensively studied and have been used most frequently to synthesize MXenes.

**Figure 13:**
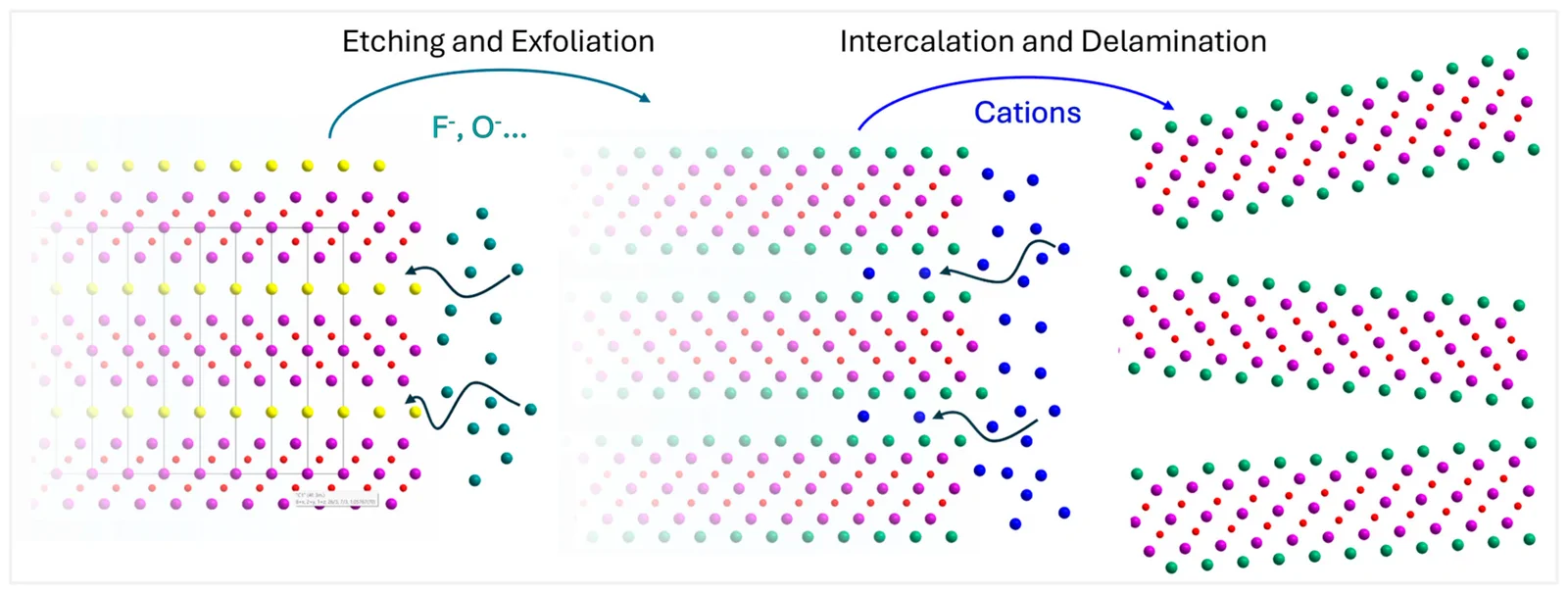
Schematic mechanism of MAX phase transformation into single-layer MXene sheets via selective etching/exfoliation of the A-element layer followed by cation intercalation and delamination.

In order to create MXenes, a selective removal of the A-layer from MAX phases is undertaken. This selective etching is possible due to the weaker M–A bonds in contrast to the stronger M–X bonds; indeed, the M–X bonds exhibit a combination of ionic, metallic, and covalent interactions, while the M–A bonds are purely metallic. This property indicates that mechanical cleavage is not an option, unlike in other layered three-dimensional (3D) materials such as graphite, which rely on weak van der Waals interactions [147,148,149,150]. MXenes follow the general formula M_n+1_X_n_T_x_ (n = 1–4), in which M is an early transition metal, X is either carbon and/or nitrogen, and Tx are surface termination groups such as hydroxyl (–OH), halogens, or chalcogens [151,152,153]. These surface terminations significantly influence hydrophilicity, etching, and material properties [154,155,156,157]. Non-MAX phases with M–A bonds can be exfoliated into MXenes as is shown in the synthesis of Mo_2_CTx from Mo_2_Ga_2_C [158]. MXenes are primarily manufactured using two main approaches: a bottom-up technique, such as chemical vapor deposition (CVD), and a top-down method encompassing various etching procedures, including electrochemical ones. Since MAX phases are the main precursors of MXenes, the etching process primarily targets their A layers (generally composed of Al or Si), which are linked by relatively weak interlayer metallic bonds compared to the robust M–X (carbide/nitride) bonds (Figure 13).

Hydrofluoric acid (HF) was the first efficient exfoliating agent of MAX phases, which efficiently removed Al from Ti_3_AlC_2_, yielding Ti_3_C_2_ MXene [159,160]. In order to move away from HF and its inherent hazards, new etching strategies have been developed. A relatively safer route with in-situ generated HF combines hydrochloric acid (HCl) and lithium fluoride (LiF). Three alternative F-free methods have been reported: (i) hydrothermal synthesis in a sodium hydroxide (NaOH) solution [161], (ii) molten salts for 2D MXene production [162,163], and (iii) electrochemical etching with HCl or ammonium chloride/tetramethylammonium hydroxide (TMAOH) electrolytes at ambient temperature [164,165]. Furthermore, MXenes were derived from Ti_3_AlC_2_ through a fluoride-free etching method that utilizes halogens (Br, I, Cl, and combinations thereof) in an anhydrous medium [166].

Thapaliya et al., however, reported a direct gas-phase fluorination method using elemental F_2_ to modify the surface chemistry of Ti_3_C_2_T_x_ (MXene) for lithium-ion battery applications [167]. In this approach, pristine MXene was carefully exposed to regulated fluorine atmospheres, allowing the embedding of fluorine while conserving the intrinsic layered structure of the material. This surface engineering enriches the surface with highly electronegative –F groups and improves resistance against oxidation and structural degradation. Although the overall layered morphology remained intact, a slight change in interlayer spacing and a moderate increase in surface disorder were detected. These structural modifications also enhanced the electronic environment by redistributing charge density across the MXene surface, which affected Li^+^ adsorption manner and diffusion pathways. In addition, the fluorinated MXene delivered improved cycling stability and higher coulombic efficiency. This work highlighted controlled fluorination as an effective technique to balance stability and performance in MXene-based energy storage systems.

## 6. Fluorination of Transition-Metal Dichalcogenides

**Figure 14:**
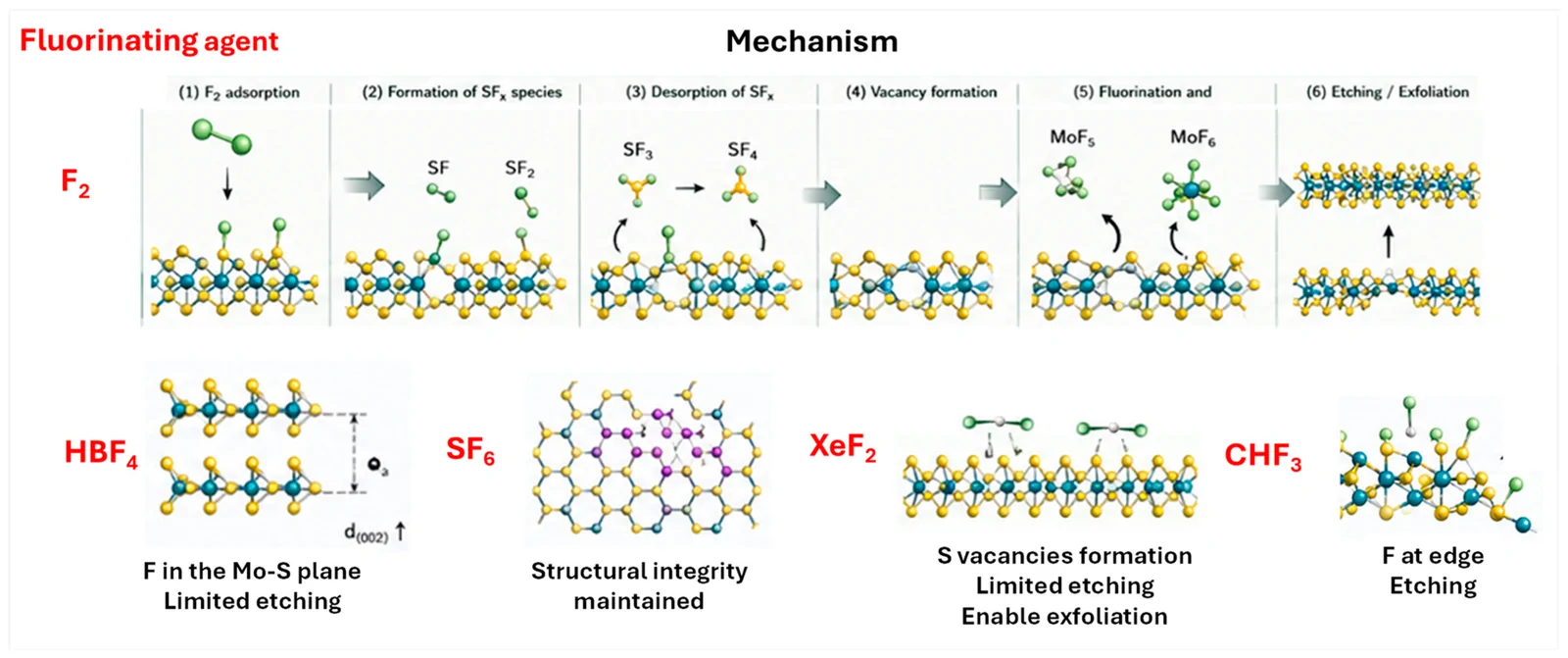
Overview of fluorination agents ${(F}_{2},\;\;{HBF}_{4},\;\;{SF}_{6},CHF_{3})$ and reaction mechanisms for ${MoS}_{2}$, showing adsorption, vacancy formation, volatile species desorption, structural modification, and etching/exfoliation.

Fluorination of TMDs, particularly MoS_2_, has mainly been explored as a strategy to tailor their structural, electronic, and electrocatalytic properties (Figure 14 and Table 2). To date, concerning MoS_2_ fluorination, several fluorination routes have been investigated, including solvothermal/hydrothermal synthesis [168,169], plasma treatments [170,171], and gas–solid fluorination using XeF_2_ [172] or F_2_ [173,174]. Wet-chemical and plasma approaches generally introduce only low fluorine contents (<10 at.%), leading to limited structural modifications while still improving the hydrogen evolution reaction (HER) activity. These improvements mainly arise from electronic effects, such as modulation of the hydrogen adsorption free energy (ΔG_H*_), rather than from significant changes in the density of active sites. Hydrothermal fluorination with NH_4_F enables simultaneous N/F co-doping, introducing 0.1–3.6 at.% fluorine without altering the characteristic rose-like morphology of MoS_2_ [168]. DFT calculations revealed that fluorine activates N-doped basal planes by decreasing ΔG_H*_ from −1.539 to −0.145 eV, thereby markedly enhancing the intrinsic activity of the existing active sites. In contrast, fluorination with HBF_4_ introduces only ~0.6 at.% fluorine, producing slight interlayer expansion and limited etching, with fluorine proposed to be incorporated within the Mo–S planes rather than only at the surface [169]. Plasma fluorination provides a versatile route to controlled surface modification. CHF_3_ plasma introduces approximately 5 at.% fluorine while simultaneously creating additional edge sites, resulting in improved HER kinetics through weaker Mo–H interactions and facilitated H_2_ desorption [171]. SF_6_ plasma treatment produces substitutional fluorine concentrations of 2–4.9 at.% and induces *p*-type doping by reducing the electron carrier density, while largely preserving the structural integrity of monolayer MoS_2_ [170]. Overall, plasma treatments primarily modify the electronic structure through defect engineering and carrier-density control rather than inducing major structural transformations. In contrast, gas–solid fluorination with XeF_2_ and especially F_2_ remains largely underexplored despite its potential to achieve significantly higher fluorine contents. Only two studies have employed XeF_2_ [172,175], and three have investigated F_2_ fluorination, all of the latter being theoretical [173,174]. XeF_2_ fluorination promotes sulfur-vacancy formation and partial Mo oxidation without severe etching, while the generated vacancies facilitate subsequent halogen incorporation and provide a favorable balance between doping and structural preservation [172]. Huang et al. proposed that the reaction proceeds mainly through the formation of volatile sulfur fluorides, enabling exfoliation of MoS_2_ into nanosheets [175]. Theoretical studies of F_2_ fluorination predict a much more extensive modification of MoS_2_ [173]. Molecular F_2_ spontaneously dissociates on the MoS_2_ surface in an exothermic process, with fluorine preferentially substituting sulfur atoms. Increasing fluorine coverage induces lattice distortions, elongation of adjacent Mo–S bonds, reduction of the electronic band gap, and a Fermi-level shift of approximately 0.61 eV [173]. Coulombic repulsion between neighboring fluorine atoms causes tilting of S–F bonds and local structural distortions, while high fluorine coverages may even trigger the 2H-to-1T phase transition [176]. Simulations further predict the formation of volatile SF_x_ (SF_2_, SF_3_, SF_4_) species followed by MoF_6(g)_, providing a plausible etching mechanism during fluorination [174]. However, these studies are restricted to mono- or bilayer MoS_2_, and the fluorination behavior of multilayer materials remains essentially unknown. 

Overview of the fluorination routes of MoS_2_ and their effects on the structure, electronic properties, and reaction mechanisms.

The literature shows that the catalytic performance of MoS_2_ arises from a complex interplay between crystal structure, defect chemistry, and electronic properties. While exfoliation, nanostructuring, and conventional defect engineering primarily increase the density of active sites, fluorination emerges as a multifunctional strategy capable of simultaneously tuning the local electronic structure, hydrogen adsorption energetics, and charge transport. Most experimental studies concerning fluorination rely on hydrothermal or plasma-based fluorination, leading to relatively low fluorine contents (<10 at.%). In contrast, gas–solid fluorination using F_2_ or XeF_2_, which could induce higher fluorine concentrations and more extensive structural modifications, remains largely unexplored. On this last point, another major limitation concerns the predominance of theoretical studies over experimental ones.

Very few examples of fluorination of other TMDs are reported in the literature, and among them, few are experimental studies. In WS_2_, increasing fluorine concentration stabilizes the metallic 1T polymorph over the semiconducting 2H phase, accompanied by modifications of the electronic structure, interlayer spacing, and tribological properties [181]. Here, the synthesis was carried out by using Nafion as the precursor for fluorine, through its thermal degradation, giving fluorine-free radicals. Fluorination of monolayer WS_2_ was achieved by fluorine plasma treatment in an inductively coupled plasma (ICP) reactor, although the fluorine precursor was not specified. Surface fluorination remained limited, reaching ~7.2% fluorine coverage. Despite this low incorporation level, fluorination markedly reduced the electron density, resulting in a ~4.2-fold enhancement of photoluminescence. It also enabled sensitive NH_3_ detection down to 1.25 ppm, whereas pristine WS_2_ showed negligible response. Notably, the process was reversible through hydrogen plasma treatment, which removed fluorine species and restored the initial electronic and optical properties [182]. The effect of fluorination on a ReS_2_ monolayer as a function of the fluorine adsorption site was investigated using first-principles DFT calculations. This study shows that when fluorine atoms are adsorbed above the Re chains, they induce metallic states within the band gap and a robust magnetic order—ferromagnetic along the Re chains and antiferromagnetic between them—whereas the adsorption of fluorine atoms between the chains leads to nonmagnetic semiconductor states. These results suggest that fluorinated ReS_2_ is a promising material for spintronic applications due to its anisotropic conduction along the Re chains [183]. Another study, based on first-principles calculations, focused on the stability and the electronic properties of monolayer-fluorinated transition metal disulfide compounds 1T-MS_2_F_2_ (M = Cr, Hf, Co and Fe), with different metallic behaviors [184].

## 7. Fluorination of Layered Double Hydroxides

LDHs, also known as hydrotalcite-type anionic clays, are a class of materials with a unique 2D layered structure and adjustable compositions and morphologies. The structure of LDHs consists of superimposed sheets made up of octahedral M(OH)_6_ units, the centers of which are occupied by divalent M^2+^ cations and the vertices by OH^−^ ions. These units share edges to build planar sheets that stack along the c-axis, resulting in a hexagonal crystallographic structure. In LDH compounds, a fraction of the divalent metal element is substituted by a trivalent metal, resulting in an excess of positive charges. To compensate for this excess charge, hydrated anions insert themselves into the interlayer spaces, rendering the mineral structure electrically neutral. Thus, the general formulation of LHD phases highlights two distinct parts: the [M^2+^_1−x_ M^3+^_x_ (OH)_2_]^x+^ layer: where M^2+^ are divalent metal cations, while M^3+^ is a metal cation often based on (Al^3+^ and Fe^3+^) and the [A^n−^_x/n_ yH_2_O] interlayer domain: where A is the intercalated anion, with n, its charge and y the number of interleaved water molecules. The ability to exchange anions gives LDHs their full range of insertion chemistry, known in various applications, including environment, catalysis, energy, and health, as recently reviewed [185,186].

We can distinguish two main effects of adding fluorine to LDHs: topotactic insertion of fluorine when added at room temperature and a transformation of the LDH template when fluorine addition is accompanied by heat treatment. In this second case, the structure changes radically; this is also the least studied case. The study of these two methods provides information on the reactivity of fluorine with LDHs as well as the change of properties of the resulting compounds.

Using a soft treatment, Xian et al. [187] prepared an LDH coated with perfluoro-octyl-tri-ethoxy-silane (PFOTES). Experimentally, LDHs-PFOTES were synthesized at room temperature, with the addition of acetic acid as a catalyst to graft PFOTES onto the positively charged surface of LDHs. This grafting is characterized mostly by XPS (Si2p) as a covalent bonding. The coated LDH exhibits a similar structure to the non-treated LDH, showing a weak hydrophobic character that is helpful in the removal of per- and polyfluoroalkyl substances (PFASs) from aqueous solutions.

Chen et al. [188] initially synthesized a carbonated CuZnAl LDH. Fluorine was then introduced using LDH reconstruction: the material was heated to 500 °C under N_2_ to deconstruct the LDH structure, which was then reformed in aqueous solution in the presence of NaF. The fluorine substituted some of the interlayer carbonate ions without altering the LDH structure. An increase in crystallinity was even observed during the reformation of the template. However, the introduction of this fluorine prevents the complete precipitation of Cu^2+^ in the newly formed LDH structure, and the appearance of a secondary CuO phase was observed at room temperature. Furthermore, the addition of fluorine resulted in a significant improvement in the compound’s thermal stability compared to the same carbonated LDH; this is explained by the strengthening of the hydrogen bonds formed with the interlayer water molecules. X-ray photoelectron spectroscopy (XPS) analyses of the calcined LDH also revealed a sample surface composed of fluorinated metals. However, these fluorinated metal phases are too amorphous and too thin to be identified by XRD. This indicates that heat treatment of the fluorinated LDH results in the formation of analogous fluorides, at least on the surface of the calcined sample.

**Figure 15:**
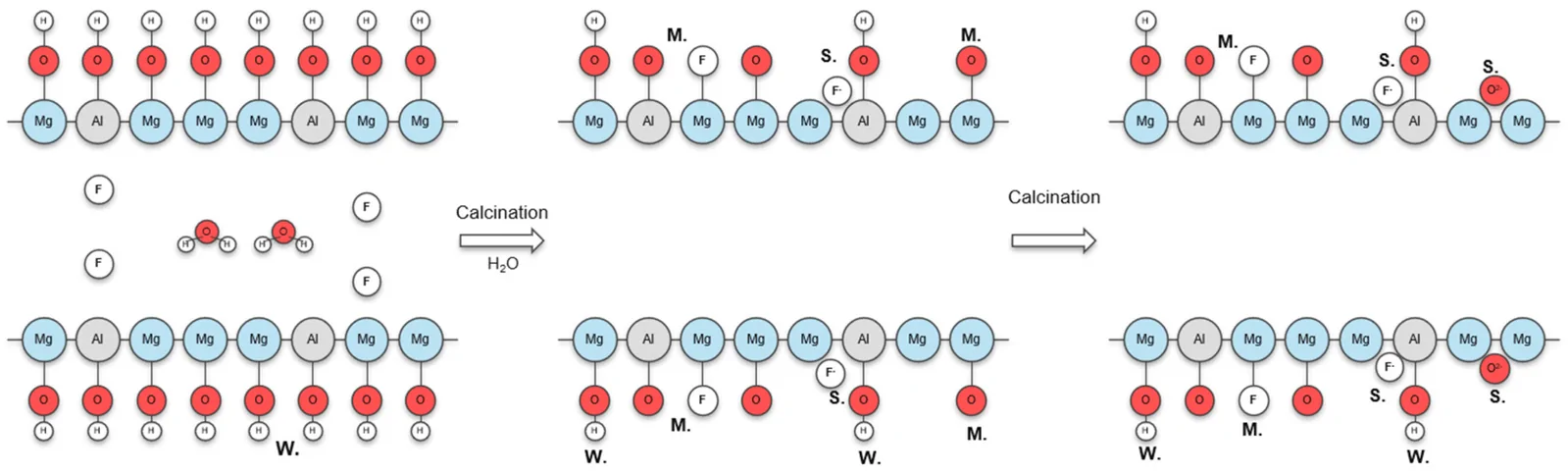
Schematic representation of the stepwise fluorination and structural transformation of Mg–Al LDH sheets during calcination illustrated in [190] with different basic sites (W.: weak; M.: moderate; S.: strong).

Depending on the metals in the LDH, the effect appears to differ. Indeed, in the work of Yang et al. [189] on a fluorine-modified MgAl LDH, also prepared by "reconstruction" in the presence of NH_4_F, the appearance of a MgF_2_ phase around 400 °C, distinguishable by XRD, was observed. A proposed evolution of the fluorinated LDH during calcination, illustrating the progressive loss of hydroxyl groups and the development of different Mg–O, Mg–F and Al–O environments and coordinatively unsaturated O^2−^ and F^−^ sites was discussed by Wu et al. [190] and illustrated in Figure 15.

Here we find a behavior analogous to that observed for Cl^−^ by Constantino et al. [191]: when some of the hydroxides leave, the interlamellar F^−^ ions take their place in the cationic sheets or position themselves in the vicinity of the metal ions, causing the formation of a secondary MgF_2_ phase observed by XRD. A lamellar structure is nevertheless preserved and results in the formation of Lamellar Double Oxides locally modified by fluorine.

Unlike the previous studies based on fluoride intercalation or reconstruction, Lima et al. introduced fluorine directly into the cationic sheets during coprecipitation by using Na_3_AlF_6_ as the aluminum source. Under controlled pH conditions, aluminum remained as octahedral (AlF_6_)^3−^ complexes, allowing the partial substitution of structural hydroxyl groups within the brucite-like layers. XRD together with ^19^F and ^27^Al MAS NMR confirmed that the LDH structure was preserved while fluorine was incorporated into the hydroxide layers rather than the interlayer space. Increasing fluorine incorporation progressively reduced the basal spacing, which was attributed to stronger interactions between the fluorinated layers, interlayer water molecules, and carbonate anions. Fluorination also modified the thermal behavior of the LDHs by shifting the dehydroxylation/decarbonation step towards higher temperatures. Moreover, calcination generated fluorinated mixed oxides with an increased proportion of coordinatively unsaturated Al(IV) sites, resulting in enhanced Lewis acidity and modified acid–base properties [192].

In a subsequent study, the same authors investigated the consequences of fluorination on the adsorption properties of these materials. They reported fluorinated MgAl LDHs with compositions up to Mg_0.714_Al_0.248_(OH)_1.59_F_0.41_(CO_3_)_0.124_·0.49H_2_O, confirming the progressive contraction of the basal spacing with increasing fluorine content. More importantly, fluorination significantly modified the surface properties of the resulting mixed oxides. Dye adsorption and ^129^Xe NMR experiments demonstrated an increase in the hydrogen-bond-accepting character, dipolarity, and polarizability of the surface, while hydroxynaphthol blue adsorption showed that fluorination progressively shields Mg^2+^ sites, thereby modifying adsorption behavior. These changes make fluorinated LDHs attractive materials for adsorption and catalytic applications [193].

**Figure 16:**
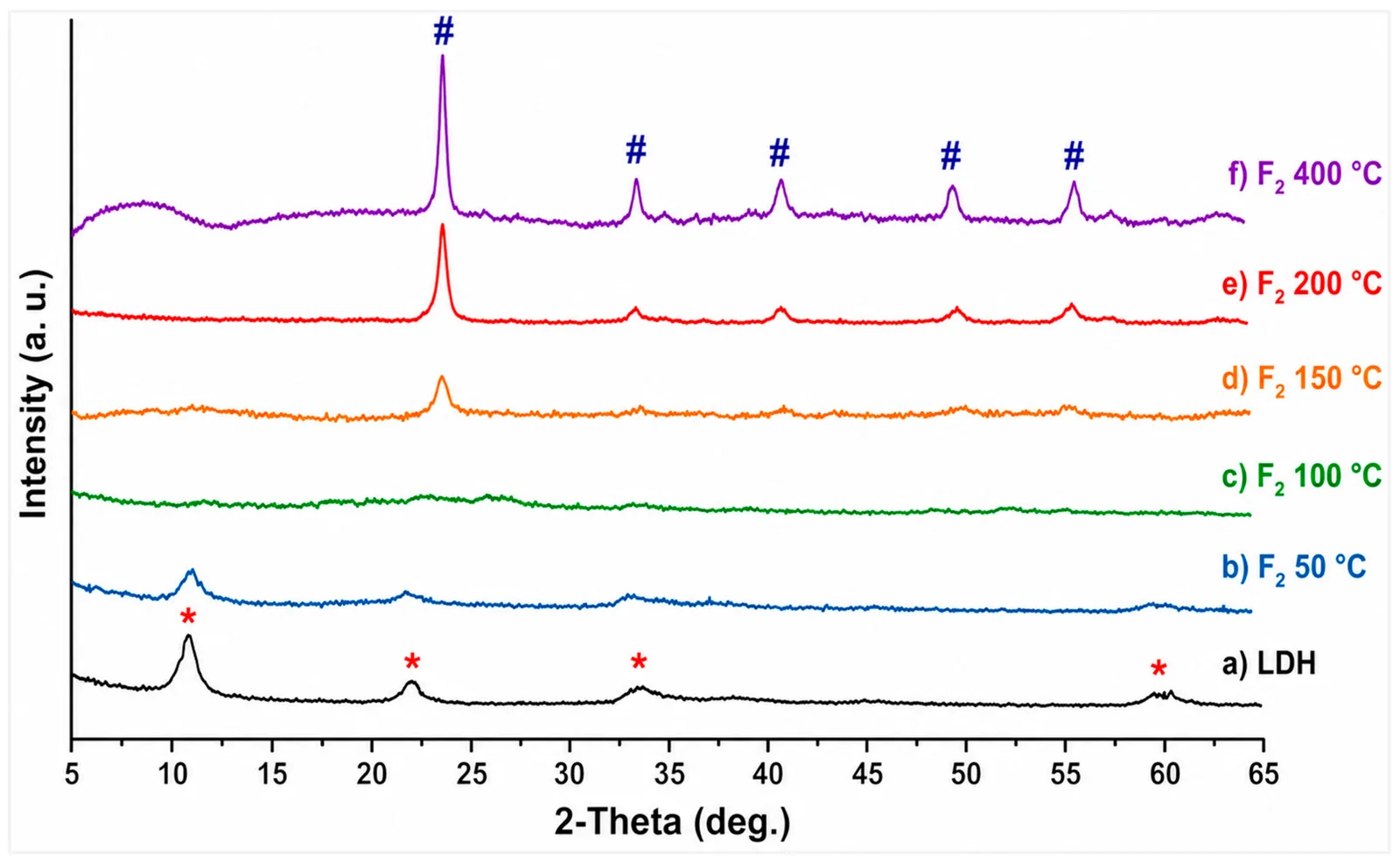
X-ray diffractograms of LDH CoFe chosen by Louvain et al. [194] and of its fluorination products at different temperatures. Asterisks * correspond to diffraction lines attributed to the LDH structure, while sharps # correspond to diffraction lines attributed to the rhombohedral ${FeF}_{3}\;({r\;-FeF}_{3})$ structure.

Regarding the molecular fluorination of LDH, Louvain et al. [194] studied the fluorination mechanism of an LDH with the composition Co_2_FeCl(OH)_6_·2H_2_O. In their protocol, fluorine was injected directly into the reactor at room temperature as well as at different temperatures. They observed that the LDH structure disappeared from the X-ray diffractogram at 100 °C under fluorine, while from 150 °C onward, a solid solution of Co_0.6_Fe_0.4_F_3_, close to the initial Co/Fe metal ratio in the LDH, gradually formed. The X-ray diffractograms of the LDH fluorination products obtained at different temperatures are shown in Figure 16.

Thus, according to this literature review, the reactivity of molecular fluorine very rapidly destabilizes the LDH structure with a temperature increase, leading to the formation of the corresponding metallic fluorides. A clear trend emerges: at room temperature and low temperatures, fluorine locally substitutes itself within the LDH structure while maintaining the typical template structure. Conversely, above a certain transition temperature, the formation of analogous fluorides is favored and is accompanied by a sharp decrease in the crystallinity of the original LDH phase.

Prepared by the same synthetic route (Na_3_AlF_6_ substitution for AlCl_3_), a series of fluorinated LDHs were tested for the photocatalytic conversion of CO_2_ [195]. The incorporation of AlF_6_^3−^ units in Ni_3_Al improves the photocatalytic conversion of CO_2_ to the reduced CO significantly. This is explained by a better adsorption of CO_2_ molecules at the surface of the fluorinated LDH, thus resulting in a selective conversion to CO.

For energy applications, fluorinated LDH-derived materials are mainly investigated as positive electrodes for lithium and lithium-ion batteries. Their interest is linked to the strong inductive effect of fluorine, since M–F bonds are more ionic than M–O bonds and can increase the redox potential of transition-metal-based electrode materials. In addition, transition-metal fluorides such as FeF_3_ and CuF_2_ can operate through conversion reactions, providing high theoretical capacities compared with classical insertion-type cathode materials. A first approach was based on Cu/Fe-containing LDHs stabilized by Mg^2+^. In this system, Mg^2+^ was required to preserve the layered LDH structure, mainly because the Jahn–Teller distortion associated with Cu^2+^ makes pure Cu-rich LDHs difficult to stabilize. After gas–solid fluorination, this precursor led to a composite mixture containing MgF_2_, CuF_2,_ and FeF_3_. Although MgF_2_ is electrochemically inactive, CuF_2_ and FeF_3_ are active toward conversion reactions with lithium. This LDH-derived mixture showed improved electrochemical behavior compared with a simple ball-milled mixture of the corresponding fluorides, highlighting the benefit of using LDHs as multi-metallic templates to promote intimate mixing of the active phases [196].

**Figure 17:**
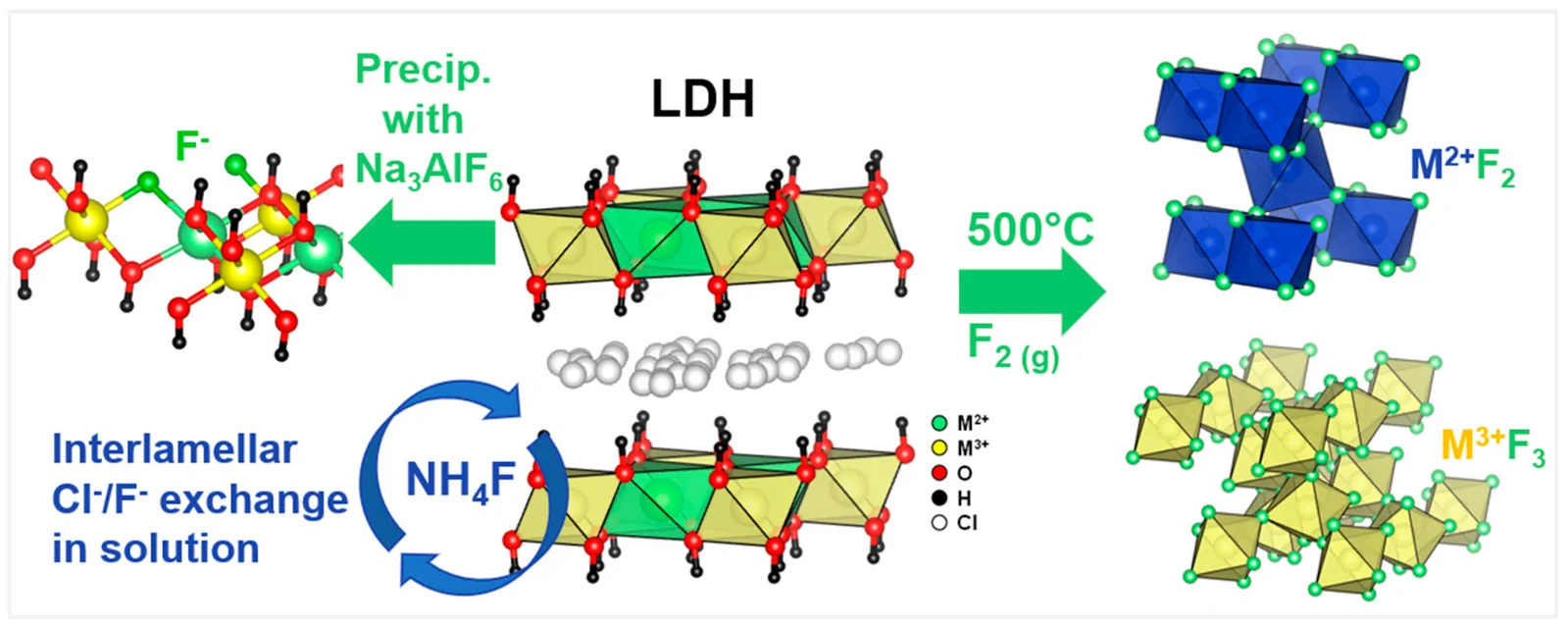
Main fluorination pathways for LDHs: intralamellar F^−^ insertion during precipitation, solution-based anion exchange (Cl−F−), and thermal gas-phase conversion into metal fluorides $(M^{2+}F_{2}$,$\;M^{2+}F_{3})$.

A second, more optimized approach consisted of replacing part of the electrochemically inactive Mg^2+^ with redox-active Co^2+^ and introducing Al^3+^ as a stabilizing cation in the trivalent layer. This led to Cu-Co-Fe-Al LDH precursors, designed to better balance structural stability and electrochemical activity after fluorination. The final products strongly depended on the treatment temperature, from amorphous or poorly crystallized porous fluorinated phases at low temperature to multiphasic fluoride composites at higher temperature, including CuF_2_, FeF_3_-type phases, and mixed Co/Fe or Al/Fe fluorides. ^57^Fe Mössbauer spectroscopy confirmed the formation of complex Fe^3+^ fluorinated environments, more distributed than in pure rhombohedral FeF_3_, due to the coexistence of several trivalent cations such as Fe^3+^, Co^3+^ and Al^3+^ within FeF_3_-type phases [197]. Overall, LDH fluorination appears as a versatile strategy, able either to introduce fluorine as interlamellar or intralamellar anions while preserving part of the layered structure, or to fully convert the LDH into intimate mixtures of metal fluorides, depending on the reaction conditions and fluorine source (Figure 17). This tunability, combined with the possibility of selecting the initial cationic composition, makes LDHs powerful precursors for designing fluorinated materials adapted to any targeted applications.

## 8. Fluorination of Intrinsically Layered Oxide Materials: V_2_O_5_ and δ-MnO_2_

Among oxide-based 2D materials, only intrinsically layered oxides are considered here. The most relevant examples are orthorhombic V_2_O_5_ and birnessite-type δ-MnO_2_. Both materials contain extended metal–oxygen slabs or sheets, but their fluorination chemistries differ markedly from the radical C–F bond formation described for graphene. In layered oxides, fluorination is better described as anion, defect, surface, and interlayer engineering.

V_2_O_5_ is a representative layered oxide benchmark. Huo et al. reported a fluorine-assisted route to transform microcrystalline V_2_O_5_ into high-purity nanosized V_2_O_5_ [198]. The fluorinating medium is aqueous HF. Micro-V_2_O_5_ is dissolved in an HF-containing aqueous solution; the solution is slowly evaporated, and the resulting solid is heat-treated at 230 °C. The final product is not described as a stable bulk V_2_O_5_^−^_x_F_x_ phase, but as nanocrystalline V_2_O_5_. Therefore, the safest mechanistic interpretation is fluorine-assisted nanostructuring rather than persistent lattice fluorination. HF likely forms transient vanadium oxyfluoride or fluorovanadate intermediates, which subsequently undergo hydrolysis, defluorination, and recondensation during thermal treatment, as summarized in the simplified reaction Equations (5) and (6):

V_2_O_5_ + 2 HF ⇌ 2 VO_2_F + H_2_O (5)

Followed by:

2 VO_2_F + H_2_O ⇌ V_2_O_5_ + 2 HF (6)

**Figure 18:**
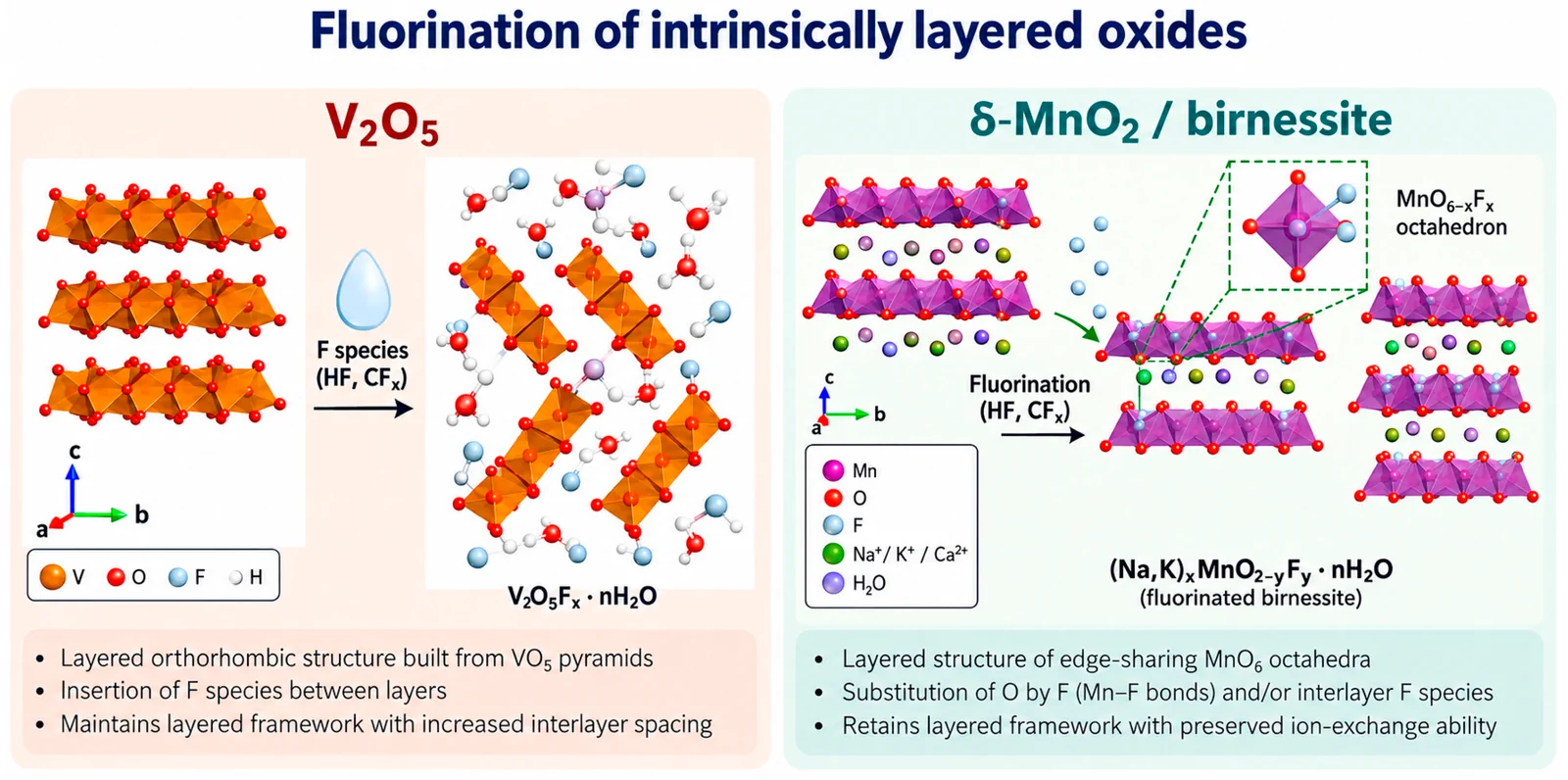
Fluorination of intrinsically layered oxides: $V_{2}O_{5}$ undergoes HF-assisted structural reconstruction, forming nanoscale V–O–F species through fluorine-induced layer disruption and progressive nanosizing. In contrast, ${\delta -MnO}_{2}$ interacts with fluoride precursors via ion-exchange or molten-salt routes, allowing interlayer fluoride incorporation and stabilization of F-retaining layered structures. Mutually, these processes control metal–fluorine bonding and interlayer redox chemistry, leading to tunable structural evolution and tailored electronic properties.

These equations should be considered as schematic balances, not as proof of an isolated VO_2_F intermediate. The main impact of the fluorine route is the formation of porous nanosized V_2_O_5_ particles, shorter Li^+^ diffusion pathways, improved electrode/electrolyte contact, and enhanced rate capability (Figure 18) [198].

A complementary kinetic study on the same nanosized V_2_O_5_ showed that the fluorine-derived nanostructuration strongly improves Li insertion kinetics [199]. The synthesis again relies on dissolution of micro-V_2_O_5_ in aqueous HF, slow evaporation, and thermal treatment at 230 °C under dynamic vacuum. Electrochemical impedance spectroscopy performed over a broad composition and temperature range showed a much lower activation energy barrier for lithium diffusion in nanosized V_2_O_5_, around 0.06 eV. This paper therefore confirms that fluorine chemistry primarily acts as a synthetic route to a kinetically improved lamellar oxide, rather than as a conventional doping strategy [199].

The high sensitivity of V_2_O_5_ toward HF/F^−^ species is further supported by electrochemical aging studies in LiPF_6_-based electrolytes [200]. In that case, HF is generated in situ by electrolyte decomposition or hydrolysis, and reacts with V_2_O_5_ during cycling. Ex situ XPS and Raman spectroscopy reveal much higher fluorine content on V_2_O_5_ cycled in LiPF_6_-containing electrolyte than in LiClO_4_-based electrolyte. This process is detrimental because it is associated with the formation of fluoride-rich or oxyfluoride-like surface species and active mass loss. Thus, V_2_O_5_ can undergo either useful fluorine-assisted nanostructuring under controlled synthesis conditions or parasitic fluorination under electrochemical aging conditions [200].

Birnessite-type δ-MnO_2_ represents a different layered oxide case because fluorine can remain chemically associated with the material. Wang et al. reported highly oriented 2D δ-MnO_2_ nanosheets containing massive anionic fluorine substitution [201]. The method is described as a lava method followed by quenching, i.e., a molten-salt/quenching route. The exact fluoride salt used as a fluorinating agent must be verified from the full experimental section, but the reported mechanism is based on anionic F incorporation, Mn–F bond formation, and stabilization of the MnO_6_ octahedral framework. Fluorine substitution also modulates the Mn^3+^/Mn^4+^ ratio by increasing the Mn^3+^ content, which is proposed to improve ion transport and electronic conductivity. When used as a zinc-ion battery cathode, the optimized material delivers 288 mAh.g^−1^ at 100 mA.g^−1^ and retains 96% capacity after 200 cycles [201].

Li et al. reported another F-doped δ-MnO_2_ system in the form of nanoflowers composed of interspersed nanosheets [202]. The fluorination is described as a two-step strategy to introduce anionic fluoride ions into δ-MnO_2_. The key mechanistic claim is that F^−^ partially replaces O^2−^ and forms Mn–F bonds. This expands the interlayer spacing available for Zn^2+^ insertion/extraction and stabilizes the MnO_2_ framework. The experimental support includes zinc-ion battery measurements. The F-doped δ-MnO_2_ nanoflowers reach 435.1 mAh.g^−1^ at 0.1 A.g^−1^, compared with 212 mAh.g^−1^ for undoped δ-MnO_2_ [202].

Finally, Shi et al. extended the concept of F-doped δ-MnO_2_ to electrocatalytic oxygen evolution [203]. MnO_2_ nanosheets and F-MnO_2_ were prepared through hydrothermal and ion-exchange methods, with control of fluorine amount and fluorination temperature. The F-doping increases the population of Mn^3+^ active centers, promotes oxygen-vacancy formation, enlarges the electrochemically active surface area, and improves alkaline OER performance. The reported overpotential is 320 mV at 10 mA cm^−2^, with stability over 25 h. This work is useful because it shows that in δ-MnO_2_, fluorine incorporation is not limited to ion-storage applications; it also tunes Mn redox chemistry and surface catalytic activity [203].

Overall, V_2_O_5_ and δ-MnO_2_ illustrate two distinct regimes of fluorination in intrinsically layered oxides. In V_2_O_5_, HF mainly behaves as a transient synthetic agent enabling dissolution–reprecipitation and nanosizing, and the final material remains V_2_O_5_. In δ-MnO_2_, fluorine can remain in the layered oxide as an anionic dopant or Mn–F environment, modifying interlayer spacing, Mn valence distribution, oxygen-vacancy concentration, and ion/electron transport. In all cases, the fluorinating agent and the final fluorine location must be explicitly identified. XPS alone is insufficient; reliable mechanistic assignment requires diffraction, Raman or IR spectroscopy, fluorine quantification, XPS of F 1 s/O 1 s/Mn or V core levels, elemental mapping, and ideally ^19^F MAS NMR or in situ/operando diffraction.

## 9. Prospects and Challenges

2D fluorinated carbons constitute a particularly promising class of materials for advanced applications in the fields of energy, tribology, and electronics, due to the possibility of finely tuning their electronic, chemical, and mechanical properties by controlling the degree of fluorination and the sp^2^/sp^3^ structure. The intensively studied case of graphene well illustrates the complexity, but also the versatility, of fluorination [16,29,30,35,37,40,42,52]. These materials offer significant application potential. In the energy sector, they are being studied as cathodes for lithium/fluorocarbon (Li/CF_x_) batteries [11,12,40,66,70,81,82,83,84,85,86,87,88], where the strong covalent C–F bond enables high energy densities, as well as components of hybrid electrodes for supercapacitors. In tribology, their low surface energy, chemical inertness, and fluorinated lamellar structure make them excellent candidates for solid lubricants and anti-wear coatings [30,45,104,112]. In electronics, the fluorination of graphene transforms a semimetal into a wide-bandgap insulator, paving the way for applications in nanoelectronics, sensors, and ultrathin insulating materials. To fully exploit this potential, several engineering strategies are being developed. Selective fluorination is a key approach aimed at spatially controlling functionalization by preferentially targeting edges, defects, or specific surface regions using masks, pre-patterns, or localized catalysts, thereby allowing for fine-tuning of the F/C ratio and the material’s local properties [48,60,94]. Another approach involves the design of hybrid materials combining fluorinated carbons with metal nanoparticles (Pt, Pd) or oxides, creating multifunctional systems that are particularly effective for electrochemical and photochemical catalysis and energy storage due to synergistic effects between the active phases and the fluorinated surface [71]. Furthermore, in line with eco-design principles, the replacement of gaseous F_2_ with less hazardous fluorinating agents such as NH_4_F or with plasma processes using CF_4_ or SF_6_ is being actively studied to reduce the environmental impact of synthesis processes. The recycling and reuse of fluorinated materials via mild defluorination processes, particularly hydrothermal or chemical ones, also represent an important avenue for limiting the ecological footprint of these materials.

The performance of fluorinated graphene, MXenes, and carbon nanosheets will largely depend on our ability to precisely control fluorination at the atomic scale. In particular, advancing these materials will require achieving a careful balance between preserving conductive sp^2^ pathways and introducing functionalized sp^3^ domains to tailor their electronic and electrochemical properties. Striking this balance is the key to maintaining rapid electron transport while leaving enough active surface sites open for chemical reactions. However, translating these materials from laboratory research to real-world applications requires addressing key challenges, including reducing conductivity loss upon fluorination, achieving uniform fluorine distribution, and developing scalable, environmentally friendly synthesis methods.

A promising emerging direction is the fluorination of heteroatom-doped carbon 2D materials, where fluorine is integrated with dopants such as boron, nitrogen, phosphorus, or oxygen to enhance material properties [7,44,64,66]. This multi-element engineering allows precise tuning of charge distribution, surface polarity, and defect chemistry, leading to significant enhancements in electrochemical energy storage. Future improvement will depend on unraveling the atomic-scale interactions between fluorine and co-dopants and understanding how their configurations govern bonding and electron transport. Such perceptions could unlock superior performance in batteries, supercapacitors, and flexible electronics, including improved capacitance, more uniform lithium deposition, and stable interfaces. However, achieving commercial feasibility still requires overcoming key barriers related to scalable production, dopant consistency, and long-term structural stability, which will rely on biomass-based precursors, eco-friendly fluorination methods, and real-time monitoring of interfacial performance.

The future of fluorinated h-BN and gC_3_N_4_ is moving beyond simple surface modification toward precise atomic-scale control of defects and interfaces [4,17,101,102,103,104,105,106,107,108,109,110,111,112,113,114,115,116,117,118,119,120,121,122,123,124,125,126,127,128,129,130,131,132,133,134,135,136,137,138,139,140,141,142]. By fine-tuning how fluorine bonds with the material, researchers can transform it from a persistent insulator into an adaptable semiconductor with magnetic or electrochemical properties. To solve this potential, the field must move toward progressive, precise synthesis methods like plasma-assisted or site-selective gas-phase processes, especially when combined with strain engineering and computational design.

The few studies on fluorination of TMDs [168,169,170,171,172,173,174,175,176,177,178,179,180,181,182,183] demonstrate that fluorine is an effective trigger for defect regulation, electronic tuning, and phase engineering in TMDs. Nevertheless, reversible fluorination–defluorination has not yet been established, and gas–solid fluorination with F_2_ or XeF_2_ remains an important yet underexplored approach for accessing higher fluorine concentrations and understanding their impact on the structure, phase stability, and electrocatalytic performance of TMDs [168,169,170,171,172,173,174,175,176,177,178,179,180,181,182,183].

Fluorination of 2D materials enables the regeneration of surface activity, the tuning of electronic and chemical properties, and sometimes the extension of the material lifetime. Moreover, fluorination weakens the interlayer van der Waals interactions in layered materials, facilitating layer separation and exfoliation into few-layer or monolayer nanosheets. These features make fluorination a promising approach for the reactivation, reprocessing, and potential recycling of 2D materials within sustainable materials engineering.

Looking forward, fluorination should be regarded not merely as a final surface modification but as an intermediate and tunable state that enables access to a broad range of functional 2D materials through controlled defluorination. The comparison of thermal, chemical, photochemical, electrochemical, and mechanochemical defluorination strategies highlights that the choice of route governs not only fluorine removal mechanisms and efficiency but also the resulting structure, surface chemistry, and functional properties. Future developments will therefore rely on tailoring defluorination pathways to achieve targeted electrical, catalytic, and energy-storage performances. Promising research directions include atmosphere-controlled defluorination, selective OH/F exchange chemistry, femtosecond-laser-assisted processing, the extension of these concepts to fluorinated nanodiamonds, and fluorine-transfer processes in composite materials. Together, these advances are expected to establish controlled defluorination as a versatile platform for engineering next-generation fluorinated materials with programmable compositions, architectures, and functionalities.

## Figures and Tables

**Table 1:** Fluorination conditions of gC_3_N_4_ and resulting properties.

Fluorinating Agent	Synthesis Method	F at.%	Structuration After Fluorination	Fluorine Atoms Positions	Specific Surface Area (m^2^/g)	Interlayer Spacing (nm)	Bandgap Energy (eV)	Applications/ Properties	Ref
[Epin]BF_4_	Post-thermal treatment	-	Nanosheet	F outside the sheet	49 (↗)	↘	↘	H_2_ production ↑ 9.5-fold (2 h)	[132]
NH_4_F	Thermal polycondensation	1.27	Quantum dot	On C atoms	-	=	↗	Bio-imaging	[133]
BminBF_4_	Thermal polycondensation	0.26	Nanosheet	On C atoms	-	0.332 (↗)	2.72 (↘)	Cyclohexane conversion ≈ 27%	[117]
NH_4_F	Thermal polycondensation	3	Nanosheet	Edge C atoms	38 (↗)	=	2.63 (↘)	H_2_ production ↑ 2.7-fold	[130]
NH_4_F	Thermal treatment	0.65	Porous nanolayers	On C	56 ↗	=	2.78 ↗	H_2_S cataluminescence detection ↑ 30-fold	[134]
NH_4_F	Thermal treatment	-	Porous nanolayers	On C atoms	-	-	1.6 (↘)	Methyl orange photodegradation ≈ 41% (40 min)	[135]
NH_4_F	Thermal treatment	-	Nanosheet	On C atoms	39.56 (↗)	↗	2.71 (↘)	MB photodegradation ≈ 96% (210 min)	[136]
NH_4_F	Co-polycondensation	0.67	Folded, irregular sheet	On C atoms	176 (↗)	=	2.82 (↘)	RhB photodegradation ≈ 96% (1 h)	[135]
NaF	Dissolution	2	Exfoliated nanolayer	On C atoms	81 (↗)	=	2.62 (↘)	H_2_ production ↑ 6-fold (3 h)	[125]
NH_4_HF_2_	Dissolution		Nanosheet	N-substitution	209 (↗)	↘	2.76 (↗)	RhB photodegradation ≈ 95% (6 min)	[119]
NH_4_F	Hydrothermal	0.3	Nano-flower Nano-stem	On C atoms	14.8 (↘)	=	↗	AO7 photodegradation ≈ 100% (3 h)	[115]
NH_4_F	Hydrothermal	0.35	Nanosheet	F-C-N-H	263 (↗)	=	2.62 (↗)	-	[118]
THF	Solvothermal	0.26	Flake	CF_2_ groups	-	=	2.97 (↗)	CO_2_ Photoreduction	[137]
Tetrafluoro terephthalic acid	Hydrothermal + calcination	0.12	Porous nanolayers	On C atoms	54.63 (↗)	↗	2.44 (↘)	CO_2_ Photoreduction	[138]
KF	Hydrothermal	2.42	Porous nanolayers	On C atoms	58.71 (↗)	-	↘	Supercapacitor capacitance ↗ by 7	[139]
BmimPF_6_	Sol-gel	1.4	Mesoporous nanolayer	On C atoms	260 (↗)	↗	1.83 (↘)	Sugar thermal conversion ≈ 91% (5 h)	[140]
F_2_/N_2_	Gas-solid reaction	7	Porous nanolayers	C atoms + interstitial F	74 (↗)	=	2.69 (↗)	H_2_ production ↑ 11.6-fold	[121]
F_2_/N_2_	Gas-solid reaction	-	Porous nanolayers	C atoms + trapped F	16 (↗)	=	-	Lithium-ion battery electrode	[120]
F_2_/N_2_	Gas-solid reaction	19	Nanosheet	C atoms + trapped/physisorbed F	7.2 (↘)	=	2.62 (↘)	MO photodegradation ≈ 93% (120 min)	[122]
XeF_2_	Gas-solid/Photochemical treatment	10.6	Quantum dot	On C atoms	-	-	3.29 (↘)	-	[141]
XeF_2_	Gas-solid/Thermal treatment	2.04	Thin nanosheets	(N_2_)CF	-	=	=	H_2_ production ↑ 14.5-fold	[123]
F	DFT calculation	7	Single-sheet	N-tripontate + C–N-tripontate	-	-	0.64 (↘)	-	[142]

**Table 2:** Reported fluorination strategies for MoS_2_, including fluorine precursors, synthesis methods, fluorine incorporation sites, fluorine contents, and resulting applications or performance improvements.

Fluorine Precursor	Fluorination Method	MoS_2_ Morphology	Fluorine Incorporation Site	Fluorine Content (at.%)	Target Application/Performance Enhancement	Ref.
**CHF_3_**	Plasma treatment	Nanosheets	Substitutional Mo sites	28.7	HER: overpotential reduced by 160 mV and Tafel slope reduced by 136 mV dec^−1^	[171]
**NH_4_F**	Hydrothermal synthesis	Flower-like microspheres	Substitutional Mo + interstitial sites	3.6	HER: overpotential reduced by 110 mV and Tafel slope reduced by 57 mV dec^−1^	[168]
**NH_4_F**	Hydrothermal synthesis	Nanosheets	Not specified	7.0	Induced ferromagnetic behavior	[177]
**HBF_4_**	Chemical synthesis	Flake-like structures	Substitutional Mo sites	0.6	Cathode material for magnesium batteries	[169]
**SF_6_**	Chemical vapor deposition (CVD)	Monolayer	Substitutional Mo sites	4.9	Flexible electronic devices	[170]
**F^−^**	Ion irradiation	Nanosheets	Substitutional Mo sites	1.95	HER: overpotential reduced by 228 mV and Tafel slope reduced by 56 mV dec^−1^	[178]
**CF_4_**	Plasma treatment	Nanosheets	Sulfur vacancies	—	Field-effect transistor (FET)	[179]
**NaF**	Hydrothermal synthesis	Nanopetals	Substitutional Mo sites	—	Zinc-ion storage electrode	[180]

## Data Availability

No new data were created or analyzed in this study. Data sharing is not applicable to this article.

## Abbreviations

The following abbreviations are used in this manuscript:

Abbreviation	Definition
2D	Two-dimensional
h-BN	Hexagonal boron nitride
TMDs	Transition metal dichalcogenides
MoS_2_	Molybdenum disulfide
WS_2_	Tungsten disulfide
LDHs	Layered Double Hydroxides
F_2_	Elemental fluorine
XeF_2_	Xenon difluoride
HF	Hydrofluoric acid/Hydrogen fluoride
NF_3_	Nitrogen trifluoride
CF_4_	Carbon tetrafluoride
SF_6_	Sulfur hexafluoride
DFT	Density Functional Theory
BF3	Boron trifluoride
XPS	X-ray photoelectron spectroscopy
NMR	Nuclear magnetic resonance
F/C	Fluorine-to-carbon ratio
Li/CF	Lithium/fluorocarbon
CVD	Chemical vapor deposition
GO	Graphene oxide
FGO	Fluorinated graphene oxide
Li^+^	Lithium ion
SEI	Solid electrolyte interphase
RF	Radio frequency
FB-LIG	Fluorine and boron co-doped laser-induced graphene
COF	Coefficient of friction
FETs	Field-effect transistors
F-Gn/F-hBN	Fluorinated graphene/Fluorinated hexagonal boron nitride
F-CNFs	Fluorinated carbon nanofibers
XRD	X-ray diffraction
